# A Multicomponent Simulation-Based Program Incorporating Ultrasound for Lumbar Puncture Training in Medical Students: A Randomized Controlled Educational Trial

**DOI:** 10.3390/jcm15197508

**Published:** 2026-09-27

**Authors:** Amirah Alatawi, Hassan Moria, Talal Turki Alqubali, Fatma Toonisi, Masad Alanazi, Mishari Beragdar, Ahamed Bashaweeh, Majidah Saad Alanazi

**Affiliations:** 1 Department of Family Medicine, University of Tabuk, Tabuk 71491, Saudi Arabia; am.alatawi@ut.edu.sa; 2 Department of Surgery, University of Tabuk, Tabuk 71491, Saudi Arabia; talal1alqubali@gmail.com (T.T.A.); fatmatoonisi@gmail.com (F.T.); drmasadalenazi@gmail.com (M.A.); 3 Department of Surgery, Al-Baha University, Al Baha 65431, Saudi Arabia; mberagdar@bu.edu.sa; 4 Department of Anesthesiology, King Abdulaziz University, Jeddah 21589, Saudi Arabia; aabashaweeh@kau.edu.sa; 5 Ministry of Human Resources and Social Development, Tabuk Rehabilitation Center, Tabuk 47311, Saudi Arabia

**Keywords:** lumbar puncture, medical education, simulation-based education, ultrasound, procedural skills, Objective Structured Clinical Examination, randomized controlled educational trial, medical students

## Abstract

**Background:** Lumbar puncture (LP) education requires integration of anatomical knowledge, procedural skills, and safety-related decision-making. Simulation incorporating preprocedural ultrasound localization provides a structured approach to LP training; however, randomized evidence among undergraduate medical students remains limited. **Methods:** This single-center, parallel-group randomized controlled educational trial included 78 clinical-year medical students randomized to a multicomponent ultrasound-integrated simulation-based LP program (UIS-LP; *n* = 38) or self-directed learning (SDL; *n* = 40). The prespecified primary outcome was immediate post-intervention simulation-based LP procedural performance assessed using a 14-item Objective Structured Clinical Examination (OSCE; score range, 0–28). Secondary outcomes included procedural knowledge, ultrasound sonoanatomy identification, self-confidence, simulated vasovagal reaction management, and LP procedural performance at 2–4 weeks. Because of the observed baseline difference in procedural knowledge, the post-intervention procedural-knowledge comparison was evaluated using post hoc ANCOVA adjusted for baseline procedural knowledge. A separate post hoc ANCOVA adjusting for baseline procedural knowledge was conducted as a sensitivity analysis for the primary OSCE outcome. Secondary analyses were exploratory, with a post hoc Bonferroni sensitivity threshold of α = 0.0125. **Results:** Immediate OSCE scores were 24.42 ± 2.13 in the UIS-LP group and 17.29 ± 4.64 in the SDL group, with a prespecified unadjusted between-group mean difference (UIS-LP − SDL) of 7.13 points (95% CI, 5.49–8.77; Cohen’s *d* = 1.96; *p* < 0.001). The post hoc ANCOVA sensitivity analysis yielded an adjusted mean difference of 8.29 points (95% CI, 6.32–10.27; *p* < 0.001). For procedural knowledge, the baseline-adjusted means were 7.90 for UIS-LP and 4.73 for SDL (adjusted mean difference, 3.17 points; 95% CI, 2.46–3.89; *p* < 0.001). Mean between-group differences were 2.97 points for ultrasound sonoanatomy identification (95% CI, 2.30–3.64), 9.89 points for self-confidence (95% CI, 7.17–12.61), and 4.24 points for simulated vasovagal reaction management (95% CI, 3.38–5.09); all four immediate exploratory secondary comparisons remained below the post hoc Bonferroni threshold. At the 2–4-week reassessment, the between-group OSCE mean difference was 7.51 points (95% CI, 6.17–8.85; Cohen’s *d* = 2.54; *p* < 0.001), whereas the group × time interaction was not statistically significant (β = 0.38 points; 95% CI, −1.67 to 2.44; *p* = 0.714). No educational or simulation-related unintended events meeting the predefined monitoring criteria were documented. **Conclusions:** Immediate simulation-based LP procedural-performance scores were higher in the UIS-LP group than in the SDL group, with between-group differences also observed across the exploratory secondary outcomes. At the 2–4-week reassessment, OSCE procedural-performance scores were higher in the UIS-LP group; however, the group × time interaction was not statistically significant, and the reassessment should not be interpreted as evidence of differential skill retention. The findings characterize the multicomponent UIS-LP program as delivered relative to SDL and cannot be attributed specifically to ultrasound or any other individual component. Further studies should examine longer-term procedural performance and whether between-group differences observed in simulation are also present during appropriately supervised patient-based LP practice.

## 1. Introduction

Lumbar puncture (LP) is an essential diagnostic and therapeutic procedure used in the evaluation of central nervous system infections, inflammatory disorders, subarachnoid hemorrhage, and neurological malignancies, as well as for cerebrospinal fluid pressure measurement and intrathecal drug administration [1,2]. Its performance requires appropriate patient preparation, anatomical localization, aseptic technique, and needle placement [1,2].

LP can be technically challenging, and opportunities for supervised procedural practice during medical training may vary [3,4]. Simulation-based education provides opportunities for structured procedural learning, repeated practice, feedback, and assessment in a controlled educational environment [5]. Previous LP studies have reported higher post-training procedural-performance scores following simulation-based mastery learning and deliberate practice [3,6], while systematic reviews have examined simulation-based education and performance outcomes across medical and other health-professions learners [7,8].

Ultrasound can provide additional anatomical information during neuraxial procedural planning, particularly when surface anatomical landmarks are difficult to identify [9,10,11]. Preprocedural ultrasound can assist with identification of relevant lumbar anatomy, vertebral levels, and the intended puncture location [9,10,11]. Ultrasound has also been incorporated into medical and procedural education using approaches that include anatomical identification, ultrasound instruction, hands-on learning, and simulation-based procedural training [12,13,14,15]. In the present study, ultrasound was used for preprocedural localization only and not for real-time visualization or guidance of needle advancement.

Procedural education may also incorporate recognition and initial management of unexpected intraprocedural events. Vasovagal reactions can include symptoms and signs such as nausea, diaphoresis, hypotension, bradycardia, and transient loss of consciousness [16,17]. The present program included a standardized simulated vasovagal reaction requiring recognition, interruption of the procedure when appropriate, supportive measures, reassessment, communication, assistance-seeking, and escalation when indicated. More broadly, simulation-based crisis-resource-management and anesthesiology education literature has addressed structured practice involving communication, teamwork, recognition, and responses to evolving clinical situations [18,19]. The scenario in the present study represented one predefined simulated event and was not intended to indicate the relative importance of vasovagal reactions among LP-related complications.

Simulation-based LP education has been evaluated previously [3,6], but evidence specifically integrating preprocedural ultrasound localization within undergraduate LP simulation remains limited. Contemporary literature describes ultrasound and simulation-based procedural education across related settings [12,13,14,15], while the timing and duration of follow-up assessments vary across procedural-simulation studies. A systematic review examining retention of procedural skills after simulation highlights the relevance of assessing procedural performance beyond the immediate post-training period [20]. Evidence evaluating preprocedural ultrasound localization, simulated LP practice, a standardized intraprocedural event, and short-term procedural reassessment within the same undergraduate educational program remains limited.

Self-directed learning (SDL) was selected as the comparator because SDL is an established approach within health-professions education and has been evaluated using a range of educational and assessment strategies [21,22]. In the present study, SDL consisted of independent access to predefined LP educational materials, whereas UIS-LP included instructor-led teaching, supervised ultrasound scanning, hands-on simulated LP practice, repeated practice, feedback, and structured debriefing. The educational conditions were not matched for instructor contact time or hands-on procedural exposure; therefore, the comparison represents the two educational conditions as delivered rather than equivalent instructional exposures.

The present randomized controlled educational trial compared a multicomponent ultrasound-integrated simulation-based lumbar puncture (UIS-LP) program with SDL among clinical-year medical students. The prespecified primary outcome was immediate post-intervention LP procedural performance assessed using a 14-item Objective Structured Clinical Examination (OSCE). Secondary outcomes were procedural knowledge, ultrasound sonoanatomy identification, self-confidence, simulated vasovagal reaction management, and LP procedural performance at the 2–4-week reassessment. The study evaluated UIS-LP as a multicomponent educational program relative to SDL and was not designed to isolate the contribution of ultrasound or any other individual component.

## 2. Materials and Methods

### 2.1. Study Design and Setting

This prospective, single-center, parallel-group randomized controlled educational trial was conducted among clinical-year medical students at the University of Tabuk, Saudi Arabia, between 22 June and 25 August 2026. Participants were randomized in a 1:1 ratio to a multicomponent ultrasound-integrated simulation-based lumbar puncture (UIS-LP) program or self-directed learning (SDL). The study compared UIS-LP as an integrated educational program with SDL and was not designed to evaluate individual program components separately.

Training and assessments were conducted at the University of Tabuk Simulation Center using an Ultrasound Compatible Lumbar Puncture/Epidural Simulator (M43E; Kyoto Kagaku Co., Ltd., Kyoto, Japan) and a portable FUJIFILM SonoSite ultrasound system (FUJIFILM SonoSite, Inc., Bothell, WA, USA). The simulator provided sonographically identifiable lumbar anatomy and supported standardized LP practice and assessment.

The study was reported in accordance with the Consolidated Standards of Reporting Trials (CONSORT) 2025 statement.

### 2.2. Participants, Eligibility, and Recruitment

Fourth-, fifth-, and sixth-year medical students enrolled in the Bachelor of Medicine and Bachelor of Surgery (MBBS) program at the University of Tabuk were recruited through institutional announcements and direct invitations during the study period.

Students were eligible if they were enrolled in one of the specified clinical years, provided written informed consent, and were available to complete the allocated educational condition and study assessments. Exclusion criteria were previous formal simulation-based LP training, advanced ultrasound-assisted LP training, or failure to complete the baseline assessment.

Previous LP exposure during routine clinical education, defined as observation of or participation in LP in a clinical setting, was recorded as a baseline characteristic and was not an exclusion criterion. This was distinguished from the formal simulation-based or advanced ultrasound-assisted LP training specified in the exclusion criteria.

Participation was voluntary. Students received information about the study procedures, educational conditions, and assessments before enrollment, and written informed consent was obtained before any study-specific procedures.

### 2.3. Sample Size

The sample size was calculated a priori using G*Power version 3.1.9.7 for the prespecified primary outcome of immediate post-intervention LP procedural performance assessed using the Objective Structured Clinical Examination (OSCE). Based on a two-sided independent-samples *t* test, Cohen’s *d* = 0.70, α = 0.05, and 80% power, the minimum required sample size was 68 participants (34 per group). Allowing for approximately 10% attrition or incomplete primary-outcome assessment, the recruitment target was increased to 76 participants.

The calculation was based on the anticipated standardized between-group difference for the primary outcome and did not account for exploratory secondary outcomes or post hoc analyses.

### 2.4. Randomization, Allocation Concealment, and Blinding

Participants were assigned in a 1:1 ratio to UIS-LP or SDL using simple unrestricted randomization without blocking or stratification. The allocation sequence was generated using the RAND() function in Microsoft Excel by an investigator who was not involved in recruitment, delivery of the educational conditions, or outcome assessment.

Allocation was concealed using sequentially numbered, opaque, sealed envelopes prepared according to the generated sequence. Enrollment and baseline assessments were completed before the corresponding envelope was opened and group allocation was revealed.

Participants and instructors could not be blinded because the educational conditions were readily distinguishable. The two faculty assessors responsible for OSCE scoring were blinded to group allocation and were not involved in delivering either educational condition. Group assignment was not disclosed to the assessors during assessment or scoring.

### 2.5. Educational Conditions

#### 2.5.1. Ultrasound-Integrated Simulation-Based Lumbar Puncture Program

The ultrasound-integrated simulation-based lumbar puncture (UIS-LP) program consisted of a 2 h instructor-led session covering lumbar anatomy, patient positioning, aseptic technique, equipment preparation, procedural sequencing, preprocedural ultrasound localization, simulated LP performance, and management of a standardized simulated vasovagal reaction.

The ultrasound component focused on lumbar sonoanatomy and preprocedural localization. Participants identified relevant anatomical landmarks and interlaminar acoustic windows and used these findings to determine the simulated puncture level and needle-entry site. Ultrasound was used for preprocedural localization only; real-time visualization or ultrasound guidance of needle advancement was not included.

Hands-on training was conducted using the ultrasound-compatible LP simulator described in Section 2.1. Under instructor supervision, participants practiced positioning, landmark identification, ultrasound localization, sterile preparation, needle insertion, recognition of simulated cerebrospinal fluid return, and completion of the procedural sequence. Repeated practice was permitted within the scheduled session.

A standardized simulated vasovagal reaction was incorporated as a predefined intraprocedural event. Training addressed recognition of the simulated event, interruption of the procedure when appropriate, positioning and immediate supportive measures, communication and reassurance, assistance-seeking, reassessment, and escalation when indicated. Patient selection, contraindications, and other LP-related complications were included in the broader educational content but were not evaluated as separate simulated event-management outcomes.

Instructor feedback was provided during supervised practice, followed by structured debriefing. Individual hands-on training time, number of ultrasound scans, and number of simulated LP attempts were not prospectively recorded. Completion was based on participation in the scheduled session rather than achievement of a predefined proficiency threshold.

A standardized instructional framework was used across UIS-LP sessions. Sessions were delivered by instructors familiar with the study protocol, with simulation-center technical support available during training.

#### 2.5.2. Self-Directed Learning Comparator

Participants allocated to self-directed learning (SDL) received standardized educational materials addressing the predefined LP learning objectives. These comprised prerecorded instructional content, written guidance on LP technique, lumbar anatomy resources, and material addressing lumbar ultrasound sonoanatomy and preprocedural landmark identification. The assigned resources included the New York School of Regional Anesthesia (NYSORA) resource Spinal Sonography and Applications of Ultrasound for Central Neuraxial Blocks. Participants had access to the materials for 2 weeks before the immediate post-intervention assessment.

SDL did not include instructor-led teaching, supervised ultrasound scanning, hands-on LP simulation, individualized faculty feedback, or structured debriefing. Participants completed the materials independently. Individual study duration and electronic resource use were not recorded; therefore, participant-level engagement with the assigned materials could not be quantified.

Participants were instructed not to share study-specific educational materials or assessment content between groups before completion of the study assessments. Adherence to this instruction and potential cross-group contamination were not formally assessed.

The educational conditions differed in delivery format, instructor contact, supervised practice, and hands-on procedural exposure. UIS-LP was a 2 h instructor-led multicomponent session, whereas SDL provided 2 weeks of independent access to standardized educational materials and was not matched for instructor contact or hands-on procedural exposure. The comparison therefore evaluated the UIS-LP program as delivered relative to SDL and was not designed to distinguish the contribution of its individual components.

### 2.6. Outcome Measures and Assessment Procedures

The prespecified primary outcome was immediate post-intervention simulation-based lumbar puncture (LP) procedural performance, assessed using a standardized 14-item Objective Structured Clinical Examination (OSCE; score range, 0–28).

Secondary outcomes included procedural knowledge, ultrasound sonoanatomy identification, self-confidence, simulated vasovagal reaction management, and LP procedural performance at the 2–4-week reassessment. Procedural knowledge and self-confidence were assessed at baseline and immediately after the allocated educational condition. Ultrasound sonoanatomy identification and simulated vasovagal reaction management were assessed only immediately post-intervention. LP procedural performance was assessed immediately post-intervention and again at 2–4 weeks; no baseline OSCE was performed.

Each outcome was evaluated using a predefined instrument and scoring framework. The LP procedural-performance OSCE and simulated vasovagal reaction management checklist were separate measures, and the vasovagal management score was not included in the OSCE total. Complete instruments, scoring procedures, standardized prompts, behavioral anchors, and answer keys are provided in Supplementary Tables S1–S5.

#### 2.6.1. Primary Outcome: LP Procedural Performance

Immediate LP procedural performance was assessed using a standardized 14-item OSCE. Each item was scored from 0 to 2 according to item-specific behavioral anchors, yielding a total score of 0–28, with higher scores indicating more complete performance of the predefined simulation-based LP tasks. The checklist and scoring criteria are provided in Supplementary Table S4.

The primary outcome was the immediate post-intervention OSCE total score. The between-group estimate was expressed as the mean difference (UIS-LP − SDL) with a 95% confidence interval (CI), with Cohen’s *d* reported as the standardized effect size.

Two trained faculty assessors, blinded to randomized group allocation, evaluated performance using the same checklist and scoring criteria. Assessor preparation and inter-rater reliability are described in Section 2.8.

Because baseline OSCE performance was not assessed, the prespecified primary analysis compared immediate post-intervention scores between groups. Given the observed baseline difference in procedural knowledge, a post hoc analysis of covariance (ANCOVA) adjusting for baseline procedural knowledge was conducted as a sensitivity analysis (Section 2.10). The prespecified unadjusted comparison remained the primary analysis because procedural knowledge represented a cognitive outcome rather than a baseline measure of directly observed procedural performance.

#### 2.6.2. Secondary Outcomes

**Procedural knowledge.** Procedural knowledge was assessed using a 9-item instrument covering LP indications, contraindications, anatomical landmarks, procedural sequence, aseptic technique, and safety. Correct responses received 1 point, yielding a total score of 0–9. The instrument was administered at baseline and immediately post-intervention (Supplementary Table S1). Because of the observed baseline difference in procedural knowledge, the post-intervention comparison was evaluated using post hoc ANCOVA adjusting for baseline procedural knowledge. The baseline-adjusted between-group estimate was used as the principal inferential comparison for this exploratory secondary outcome (Section 2.10).

**Self-confidence.** Self-confidence was assessed at baseline and immediately post-intervention using a 10-item questionnaire rated on a five-point Likert scale. Item scores were summed to produce a total score of 10–50, with higher scores indicating greater self-reported confidence. This measure was distinct from directly observed procedural performance. The questionnaire and scoring framework are provided in Supplementary Table S2.

**Ultrasound sonoanatomy identification.** Ultrasound sonoanatomy identification was assessed immediately post-intervention using a 10-item image-based instrument addressing lumbar anatomical structures and sonographic features relevant to preprocedural LP planning. Correct responses received 1 point, yielding a total score of 0–10. The assessment evaluated recognition and interpretation of standardized ultrasound images rather than probe handling, image acquisition or optimization, real-time ultrasound-guided needle advancement, or patient-based ultrasound performance. No baseline assessment was performed. Assessment materials and scoring criteria are provided in Supplementary Table S3.

**Simulated vasovagal reaction management.** Management of a standardized simulated vasovagal reaction was assessed immediately post-intervention using a separate 7-item checklist. Each item was scored from 0 to 2, yielding a total score of 0–14. This measure was separate from the LP procedural-performance OSCE and did not contribute to its 0–28 total score. No baseline assessment was performed. The scenario, prompts, behavioral anchors, and scoring criteria are provided in Supplementary Table S5.

**LP procedural performance at 2–4 weeks.** Procedural performance was reassessed within the prespecified 2–4-week window using the same 14-item OSCE framework as the immediate assessment (Supplementary Table S4). This outcome represented simulation-based procedural performance at the reassessment point. Between-group differences in the pattern of change across the two post-intervention assessments were evaluated using the group × time analysis specified in Section 2.10. Accordingly, the reassessment score alone was not interpreted as evidence of differential skill retention.

### 2.7. Assessment Schedule and Standardization

Assessments were conducted at three predefined time points: baseline, immediately after completion of the allocated educational condition, and at the 2–4-week reassessment. Corresponding assessment procedures were applied to both randomized groups at each applicable time point.

At baseline, before randomization, participants completed the procedural knowledge assessment and self-confidence questionnaire, and demographic and educational characteristics, including previous LP exposure, were recorded. LP procedural performance, ultrasound sonoanatomy identification, and simulated vasovagal reaction management were not assessed at baseline.

Immediately post-intervention, participants completed the 14-item LP procedural-performance OSCE, procedural knowledge assessment, ultrasound sonoanatomy identification assessment, self-confidence questionnaire, and simulated vasovagal reaction management assessment. Cognitive and self-reported measures were administered separately from simulation-based performance assessments. The LP procedural-performance OSCE and simulated vasovagal reaction management assessment used separate scoring instruments, and the latter did not contribute to the 0–28 OSCE total score. The participant-level sequence of the immediate assessments was not formally recorded.

LP procedural performance was reassessed within the prespecified 2–4-week window using the same 14-item OSCE framework. The simulation environment, procedural task, equipment, candidate instructions, checklist, and scoring criteria were standardized across the immediate and reassessment time points. The other secondary outcomes were not reassessed at 2–4 weeks.

Because the same OSCE framework was used at both post-intervention assessments, testing or recall effects cannot be excluded. The 2–4-week outcome was therefore defined as simulation-based procedural performance at reassessment, with longitudinal interpretation based on the group × time analysis described in Section 2.10. Accordingly, the reassessment score alone was not interpreted as evidence of differential skill retention.

All participants completed reassessment within the prespecified 2–4-week window. Exact participant-level reassessment intervals were not retained in the analytical dataset; therefore, variation in timing within this period could not be examined.

### 2.8. Instrument Development, Validity, Reliability, and Pilot Testing

The assessment instruments were developed or adapted according to the predefined learning objectives and assessment domains and were informed by relevant literature on LP education, simulation-based assessment, ultrasound-assisted procedural education, and procedural safety. Before enrollment, the instruments underwent expert content review addressing item relevance, clarity, suitability for clinical-year medical students, and alignment with the intended assessment domains.

Where retained quantitative records were available, content validity was evaluated using the Content Validity Index (CVI). Experts rated item relevance on a four-point scale (1 = not relevant to 4 = highly relevant). The item-level Content Validity Index (I-CVI) represented the proportion of experts assigning a rating of 3 or 4, and the scale-level Content Validity Index using the average method (S-CVI/Ave) represented the mean I-CVI across items. For the 10-item self-confidence questionnaire, the documented S-CVI/Ave was 0.96, and Cronbach’s α was 0.81. Complete item-level records were not retained for all instruments; therefore, unavailable validity estimates were not retrospectively reconstructed.

Expert-review comments were incorporated before instrument finalization. Item structure, score ranges, and administration procedures were established before enrollment. The item-specific behavioral anchors presented for the LP procedural-performance OSCE and simulated vasovagal reaction management checklist in the revised Supplementary Materials provide operational descriptions of the original 0–2 scoring framework without altering the number of items, score ranges, outcome definitions, or participant-level scores.

The instruments and study procedures underwent pilot testing before formal implementation to assess clarity, feasibility, administration time, scenario flow, equipment requirements, and application of the scoring framework. Identified clarifications were incorporated before enrollment and data collection.

For the LP procedural-performance OSCE, two trained faculty assessors used the same 14-item checklist and item-specific scoring criteria. Before formal assessment, they reviewed the checklist, procedural sequence, observable performance behaviors, and application of the 0–2 scoring framework. Both assessors were blinded to randomized group allocation and were not involved in delivering either educational condition.

The available inter-rater reliability estimate for OSCE scoring was ICC = 0.89. The retained analytical dataset contained one participant-level OSCE score at each assessment point rather than separate assessor-level scores. Because the original detailed ICC output was unavailable during manuscript revision, the ICC model, agreement definition, confidence interval, and single- versus average-measure specification could not be verified and were not retrospectively reconstructed.

Complete quantitative validity and reliability estimates were not available for all secondary instruments, and unsupported coefficients were not retrospectively calculated. Instrument-specific content-review procedures, scoring frameworks, and available validity and reliability information are provided in Supplementary Tables S1–S5.

### 2.9. Educational Exposure, Contamination, and Unintended Events

Attendance and completion of the scheduled UIS-LP session were documented. Individual hands-on training time, number of ultrasound scans, and number of simulated LP attempts were not prospectively recorded; therefore, participant-level practice exposure within UIS-LP could not be quantified.

Participants allocated to SDL received the assigned standardized educational materials for independent study during the 2-week period preceding the immediate post-intervention assessment, including the designated New York School of Regional Anesthesia (NYSORA) resource Spinal Sonography and Applications of Ultrasound for Central Neuraxial Blocks. Individual study duration and electronic resource use were not recorded; therefore, participant-level engagement with SDL materials could not be quantified.

Participants were instructed not to share study-specific educational materials or assessment content between randomized groups before completion of the study assessments. Adherence to this instruction and potential cross-group contamination were not formally evaluated. Additional LP- or ultrasound-related educational exposure outside the assigned conditions was also not systematically recorded.

Educational and simulation-related unintended events were monitored during supervised training and assessment activities. Prespecified events included physical discomfort; distress or anxiety requiring interruption; equipment-related incidents; withdrawal or discontinuation related to study procedures; need for medical evaluation; or modification of planned study procedures. Participants could pause or discontinue an activity, with faculty or simulation-center personnel present throughout supervised activities.

All needle-insertion practice was performed on the designated LP simulator; **no study-related invasive procedure was performed on participants or patients**. The vasovagal reaction was a scripted simulated event presented through standardized symptoms, clinical cues, and simulated physiological findings; no actual physiological deterioration was induced.

### 2.10. Statistical Analysis

Statistical analyses were performed using IBM SPSS Statistics, version 29.0 (IBM Corp., Armonk, NY, USA). Continuous variables were summarized as mean ± standard deviation (SD) and, where appropriate, median and range; categorical variables were summarized as frequencies and percentages. All inferential tests were two-sided.

Baseline demographic, educational, and pre-intervention characteristics were summarized descriptively by randomized group without hypothesis testing. Standardized differences were used to characterize observed between-group differences. For continuous variables, standardized mean differences were calculated using the pooled SD; for binary variables, standardized differences were calculated using pooled proportional variance. An overall standardized difference was calculated for the three-category academic-year distribution.

The prespecified primary analysis compared immediate post-intervention Objective Structured Clinical Examination (OSCE) procedural-performance scores between groups using an independent-samples *t* test. Results were reported as group means ± SD, mean difference (UIS-LP − SDL) with 95% confidence interval (CI), Cohen’s *d* calculated using the pooled SD, and *p* value. Because baseline OSCE performance was not assessed, the primary analysis was based on immediate post-intervention scores.

Given the observed baseline difference in procedural knowledge, a post hoc analysis of covariance (ANCOVA) was performed as a sensitivity analysis for the primary outcome. Immediate post-intervention OSCE score was specified as the dependent variable, randomized group as the fixed factor, and baseline procedural knowledge as the covariate. Adjusted group means and the adjusted between-group mean difference (UIS-LP − SDL) were reported with 95% CI and *p* value. Because procedural knowledge represents a cognitive outcome distinct from directly observed procedural performance, this analysis did not substitute for a baseline OSCE assessment, and the prespecified unadjusted comparison remained the primary analysis.

Immediate secondary outcomes comprised procedural knowledge, ultrasound sonoanatomy identification, self-confidence, and simulated vasovagal reaction management. Because procedural knowledge showed a substantial baseline difference between groups, its post-intervention comparison was evaluated using post hoc ANCOVA, with post-intervention procedural knowledge as the dependent variable, randomized group as the fixed factor, and baseline procedural knowledge as the covariate. Adjusted group means and the adjusted between-group mean difference were reported with 95% CI and *p* value. The baseline-adjusted estimate was used as the principal inferential comparison for this exploratory secondary outcome, whereas the unadjusted post-intervention estimate was retained for descriptive transparency.

Ultrasound sonoanatomy identification, self-confidence, and simulated vasovagal reaction management were evaluated using independent-samples *t* tests of immediate post-intervention scores. Results were reported as group means ± SD, mean differences (UIS-LP − SDL) with 95% CIs, Cohen’s *d* calculated using the pooled SD, and *p* values.

For both post hoc ANCOVA models, the group × baseline procedural knowledge interaction was examined to assess the homogeneity-of-regression-slopes assumption. The interaction was not statistically significant for either procedural knowledge (*p* = 0.250) or the primary-outcome sensitivity analysis (*p* = 0.339). HC3 heteroscedasticity-consistent robust standard errors were used where heteroscedasticity was detected.

All immediate secondary-outcome analyses were exploratory. A post hoc Bonferroni sensitivity analysis was applied across the four immediate secondary outcomes using an adjusted threshold of α = 0.0125 (0.05/4). For procedural knowledge, this sensitivity assessment used the baseline-adjusted ANCOVA result; for the other three outcomes, it used the respective post-intervention between-group comparisons. The multiplicity sensitivity analysis did not alter the exploratory status of these outcomes.

OSCE procedural-performance scores at the 2–4-week reassessment were compared between groups using an independent-samples *t* test and reported as group means ± SD, mean difference (UIS-LP − SDL) with 95% CI, Cohen’s d, and *p* value. Scores across the immediate and 2–4-week assessments were additionally examined using a linear mixed-effects model with group, time, and group × time as fixed effects and participant as a random intercept. The group × time interaction was reported as β with 95% CI and *p* value. Because baseline OSCE performance was unavailable, this model evaluated score patterns across the two post-intervention assessments rather than change from baseline or differential skill retention.

Inter-rater reliability for OSCE scoring was summarized using the intraclass correlation coefficient (ICC), and internal consistency of the self-confidence questionnaire was summarized using Cronbach’s α. Available reliability information is described in Section 2.8.

Analyses used available outcome data without imputation. Statistical significance was defined as a two-sided *p* < 0.05, except for the post hoc Bonferroni sensitivity analysis of the four immediate secondary outcomes, for which α = 0.0125 was applied.

### 2.11. Ethical Considerations

The study was approved by the Research Ethics Committee of the University of Tabuk, Saudi Arabia (Approval No. UT-942-464-2026; 17 June 2026) before participant recruitment and study procedures. The study was conducted in accordance with the Declaration of Helsinki and applicable institutional ethical requirements.

Participation was voluntary, and written informed consent was obtained before enrollment and any study-specific procedures. Students were informed that participation, refusal, or withdrawal would not affect their course grades, academic assessments, progression within the MBBS program, or educational standing and that they could withdraw at any time without academic penalty.

Participant confidentiality was maintained using unique study codes. Personally identifiable information was excluded from the analytical dataset, and electronic study data were stored in password-protected files accessible only to authorized research personnel.

All study activities were conducted in an educational simulation setting. No patients were enrolled, and no invasive LP procedure was performed on students or patients. Ultrasound equipment and LP simulators were used exclusively for educational and assessment activities. After completion of all study assessments, participants allocated to SDL were offered access to the UIS-LP workshop.

There was no patient or public involvement in the design, conduct, reporting, or dissemination planning of the study.

### 2.12. Study Classification, Registration, and Data and Material Availability

This randomized educational study evaluated learner-related outcomes in medical students and did not assess health outcomes in participants or patients. It was therefore not considered a clinical trial requiring prospective registration under the ICMJE/WHO definition.

De-identified participant-level data and the corresponding data dictionary may be made available by the corresponding author upon reasonable request, subject to institutional, ethical, and data-governance requirements.

Educational and assessment materials are provided in the manuscript and Supplementary Materials. Supplementary Tables S1–S5 include the procedural knowledge assessment, self-confidence questionnaire, ultrasound sonoanatomy identification assessment, LP procedural-performance OSCE, standardized simulated vasovagal reaction management scenario, and the corresponding answer keys, behavioral anchors, and scoring criteria.

A separate statistical analysis plan was not prospectively registered or publicly archived. No public participant-level dataset or analysis-code repository was established.

## 3. Results

### 3.1. Participant Flow

Between 22 June and 25 August 2026, 84 clinical-year medical students were assessed for eligibility. Six were excluded before randomization: two had previously completed formal simulation-based lumbar puncture (LP) training, one had received advanced ultrasound-assisted LP training, two declined participation, and one did not complete the baseline assessment. The remaining 78 participants were randomized to the ultrasound-integrated simulation-based lumbar puncture (UIS-LP) group (*n* = 38) or the self-directed learning (SDL) group (*n* = 40) (Figure 1).

All 78 randomized participants received their allocated educational condition and completed the immediate post-intervention assessments and the OSCE reassessment within the prespecified 2–4-week window. There were no losses to follow-up, crossovers, or documented deviations from randomized allocation.

No educational or simulation-related unintended events meeting the predefined monitoring criteria were documented, and no participant discontinued a study activity because of an unintended event.

Participant recruitment, randomization, allocation, and follow-up are summarized in Figure 1.

### 3.2. Baseline Characteristics

Baseline demographic and educational characteristics are summarized in Table 1. The overall mean age was 22.15 ± 1.06 years; 46 participants (59.0%) were female, and 32 (41.0%) were male. Fifth-year students comprised 60.3% of the sample (47/78), followed by fourth-year students (17/78, 21.8%) and sixth-year students (14/78, 17.9%). Previous LP exposure was reported by 20 participants (25.6%).

Baseline characteristics were summarized descriptively without between-group hypothesis testing. Mean age was 21.74 ± 0.75 years in the UIS-LP group and 22.55 ± 1.18 years in the SDL group, corresponding to a standardized difference of −0.81 (UIS-LP − SDL). Standardized differences were −0.04 for female sex and −0.21 for previous LP exposure. The overall standardized difference for the academic-year distribution was 0.48 (Table 1).

Pre-intervention educational measures are presented in Table 2. Mean procedural knowledge scores were 6.08 ± 1.67 in the UIS-LP group and 3.15 ± 1.83 in the SDL group, corresponding to a standardized difference of 1.67. Mean self-confidence scores were 25.6 ± 6.0 and 27.0 ± 9.9, respectively, corresponding to a standardized difference of −0.17.

Because of the observed baseline difference in procedural knowledge, the immediate post-intervention procedural-knowledge comparison was evaluated using post hoc analysis of covariance (ANCOVA) adjusting for baseline procedural knowledge. The baseline-adjusted estimate was used as the principal inferential comparison for this exploratory secondary outcome. For the primary OSCE outcome, adjustment for baseline procedural knowledge was performed only as a post hoc sensitivity analysis; the prespecified unadjusted comparison remained the primary analysis.

Baseline OSCE procedural performance was not assessed. Accordingly, baseline comparability for directly observed procedural performance could not be evaluated, and baseline procedural knowledge should not be interpreted as a surrogate for baseline OSCE performance.

### 3.3. Baseline Educational Outcomes

Additional descriptive baseline educational results are presented in Table 2 and Table 3. Median procedural knowledge scores were 6.5 (range, 3–9) in the UIS-LP group and 3.0 (range, 1–8) in the SDL group. Mean procedural knowledge scores corresponded to 67.6% and 35.0% of the maximum possible score, respectively (Table 2).

Baseline self-confidence domain scores are presented descriptively in Table 3. Mean scores for UIS-LP and SDL, respectively, were 2.79 ± 0.66 and 2.55 ± 1.15 for anatomical landmark identification; 2.71 ± 0.57 and 2.55 ± 1.22 for patient positioning; 2.50 ± 0.76 and 2.70 ± 1.09 for procedural steps; 2.39 ± 0.97 and 2.65 ± 1.37 for contraindications and procedural safety; and 2.42 ± 0.89 and 3.05 ± 1.36 for aseptic technique. These domain-level results were descriptive, and no between-group hypothesis tests were performed.

### 3.4. Primary Outcome

#### Lumbar Puncture Procedural Performance

Immediate post-intervention lumbar puncture (LP) procedural performance is presented in Table 4. Mean Objective Structured Clinical Examination (OSCE) scores were 24.42 ± 2.13 in the UIS-LP group and 17.29 ± 4.64 in the SDL group. The prespecified unadjusted between-group mean difference (UIS-LP − SDL) was 7.13 points on the 0–28 scale (95% CI, 5.49–8.77; Cohen’s *d* = 1.96; *p* < 0.001).

A post hoc analysis of covariance (ANCOVA), conducted as a sensitivity analysis because of the observed baseline difference in procedural knowledge, yielded adjusted mean OSCE scores of 25.02 for UIS-LP and 16.73 for SDL, with an adjusted between-group mean difference of 8.29 points (95% CI, 6.32–10.27; *p* < 0.001) (Table 5). The group × baseline procedural knowledge interaction was not statistically significant (*p* = 0.339). HC3 heteroscedasticity-consistent robust standard errors were used because heteroscedasticity was detected, and residual assessment did not indicate departure from normality (Shapiro–Wilk *p* = 0.832). This post hoc ANCOVA was considered a sensitivity analysis and did not replace the prespecified unadjusted primary comparison. Because baseline OSCE performance was not assessed, adjustment for baseline procedural knowledge does not establish baseline comparability in directly observed procedural performance.

Inter-rater reliability for OSCE scoring was ICC = 0.89. The ICC model, agreement definition, confidence interval, and single- versus average-measure specification could not be verified from the retained records and were not retrospectively reconstructed.

Normalized immediate post-intervention outcomes are presented descriptively in Figure 2. Normalization was used only for graphical comparison across measures with different score ranges; all inferential analyses were conducted using the original, non-normalized scores.

### 3.5. Secondary Outcomes

Immediate secondary outcomes comprised procedural knowledge, ultrasound sonoanatomy identification, self-confidence, and simulated vasovagal reaction management (Table 4 and Table 5). These analyses were exploratory. In a post hoc Bonferroni sensitivity analysis using an adjusted threshold of α = 0.0125, all four comparisons remained below the adjusted threshold. For procedural knowledge, this sensitivity assessment used the baseline-adjusted ANCOVA result presented in Table 5; for the other three outcomes, it used the respective unadjusted post-intervention between-group comparisons presented in Table 4. This multiplicity sensitivity analysis did not alter the exploratory status of these outcomes.

#### 3.5.1. Procedural Knowledge

Mean immediate post-intervention procedural knowledge scores were 8.11 ± 0.68 in the UIS-LP group and 4.54 ± 1.68 in the SDL group (Table 4). Because of the observed baseline difference in procedural knowledge, the post-intervention comparison was adjusted for baseline procedural knowledge using post hoc ANCOVA. Adjusted means were 7.90 for UIS-LP and 4.73 for SDL, corresponding to an adjusted between-group mean difference of 3.17 points (95% CI, 2.46–3.89; *p* < 0.001) (Table 5). The group × baseline procedural knowledge interaction was not statistically significant (*p* = 0.250), and HC3 heteroscedasticity-consistent robust standard errors were used for inference. The baseline-adjusted estimate was used as the principal inferential comparison for this exploratory secondary outcome.

The unadjusted post-intervention mean difference was 3.57 points (95% CI, 2.98–4.15; Cohen’s *d* = 2.76; *p* < 0.001) and is presented in Table 4 as a descriptive supporting result.

#### 3.5.2. Ultrasound Sonoanatomy Identification

Mean immediate post-intervention ultrasound sonoanatomy identification scores were 7.75 ± 1.05 in the UIS-LP group and 4.78 ± 1.81 in the SDL group. The between-group mean difference was 2.97 points on the 0–10 scale (95% CI, 2.30–3.64; Cohen’s *d* = 1.99; *p* < 0.001) (Table 4).

This assessment was administered only after the educational conditions and evaluated recognition and interpretation of standardized lumbar ultrasound images. It did not assess probe handling, image acquisition or optimization, real-time ultrasound-guided needle advancement, or patient-based procedural performance.

#### 3.5.3. Self-Confidence

Mean immediate post-intervention self-confidence scores were 39.60 ± 4.40 in the UIS-LP group and 29.71 ± 7.24 in the SDL group, corresponding to a between-group mean difference of 9.89 points on the 10–50 scale (95% CI, 7.17–12.61; Cohen’s *d* = 1.64; *p* < 0.001) (Table 4).

Baseline self-confidence scores were 25.6 ± 6.0 in the UIS-LP group and 27.0 ± 9.9 in the SDL group (standardized difference, −0.17; Table 2), with domain-level descriptive results presented in Table 3. Self-confidence was a self-reported outcome distinct from directly observed procedural performance.

#### 3.5.4. Simulated Vasovagal Reaction Management

Mean immediate post-intervention simulated vasovagal reaction management scores were 11.83 ± 1.41 in the UIS-LP group and 7.59 ± 2.26 in the SDL group. The between-group mean difference was 4.24 points on the 0–14 scale (95% CI, 3.38–5.09; Cohen’s *d* = 2.24; *p* < 0.001) (Table 4).

This assessment was separate from the 14-item LP procedural-performance OSCE and did not contribute to its 0–28 total score. It was administered only after the educational conditions and was limited to performance within the standardized simulated vasovagal scenario.

#### 3.5.5. Normalized Immediate Outcomes

For descriptive graphical comparison, immediate post-intervention mean scores were expressed as percentages of their respective maximum possible scores (Figure 2). The SDL and UIS-LP values were 61.8% and 87.2% for OSCE procedural performance, 50.4% and 90.1% for procedural knowledge, 47.8% and 77.5% for ultrasound sonoanatomy identification, 59.4% and 79.2% for self-confidence, and 54.2% and 84.5% for simulated vasovagal reaction management, respectively.

Normalization was used only for descriptive graphical presentation. All inferential analyses were conducted using the original, non-normalized scores. For procedural knowledge, the baseline-adjusted ANCOVA estimate presented in Table 5 served as the principal inferential comparison and is not represented by the normalized values in Figure 2.

#### 3.5.6. Procedural Performance at the 2–4-Week Reassessment

At the 2–4-week reassessment, mean OSCE procedural-performance scores were 23.45 ± 2.59 in the UIS-LP group and 15.94 ± 3.28 in the SDL group. The between-group mean difference was 7.51 points on the 0–28 scale (95% CI, 6.17–8.85; Cohen’s *d* = 2.54; *p* < 0.001) (Table 6).

Mean scores changed from 24.42 ± 2.13 at the immediate assessment to 23.45 ± 2.59 at the 2–4-week reassessment in the UIS-LP group and from 17.29 ± 4.64 to 15.94 ± 3.28, respectively, in the SDL group. The group × time interaction was not statistically significant (β = 0.38 points; 95% CI, −1.67 to 2.44; *p* = 0.714), indicating no statistical evidence of a between-group difference in the pattern of score change across the two post-intervention assessments.

Accordingly, the 2–4-week result represents a between-group difference in procedural performance at reassessment rather than evidence of differential skill retention. Baseline OSCE performance was not assessed, and the same OSCE framework was used at both post-intervention assessments; therefore, change from pre-intervention performance and potential testing or recall effects could not be evaluated.

## 4. Discussion

### 4.1. Principal Findings

The prespecified primary analysis showed higher immediate simulation-based lumbar puncture (LP) procedural-performance scores in the UIS-LP group than in the SDL group, with an unadjusted between-group mean difference of 7.13 points (95% CI, 5.49–8.77; Cohen’s *d* = 1.96; *p* < 0.001). Previous LP education studies have similarly reported higher post-training procedural-performance scores following simulation-based mastery learning and deliberate practice [3,6]. A post hoc sensitivity analysis adjusting for baseline procedural knowledge showed a between-group difference in the same direction (adjusted mean difference, 8.29 points; 95% CI, 6.32–10.27; *p* < 0.001). However, baseline procedural knowledge represents cognitive performance rather than directly observed procedural performance and cannot substitute for the unavailable baseline OSCE assessment. Accordingly, the prespecified unadjusted comparison remained the primary analysis.

The exploratory secondary outcomes showed between-group differences across several educational domains. Because procedural knowledge differed substantially between groups at baseline, the baseline-adjusted post-intervention estimate was used as the principal inferential comparison for this outcome (adjusted mean difference, 3.17 points; 95% CI, 2.46–3.89; *p* < 0.001). Higher immediate post-intervention scores were also observed in the UIS-LP group for ultrasound sonoanatomy identification, self-confidence, and simulated vasovagal reaction management. These outcomes represent distinct constructs—cognitive performance, image-based anatomical recognition, self-reported confidence, and performance within a standardized simulated event—and should not be interpreted as interchangeable indicators of procedural competence. The sonoanatomy finding can be considered in the context of literature describing ultrasound and simulation-based procedural education [12,13,14,15]; however, the assessment was image-based and administered only post-intervention and therefore does not establish an independent contribution of ultrasound, practical ultrasound proficiency, or transfer beyond the assessed task.

At the 2–4-week reassessment, OSCE procedural-performance scores were higher in the UIS-LP group than in the SDL group (mean difference, 7.51 points; 95% CI, 6.17–8.85; *p* < 0.001). However, the group × time interaction across the immediate and 2–4-week assessments was not statistically significant (β = 0.38 points; 95% CI, −1.67 to 2.44; *p* = 0.714), indicating no statistical evidence of a between-group difference in the pattern of score change across the two post-intervention assessments. Accordingly, the reassessment demonstrates a between-group difference in procedural performance at 2–4 weeks but does not provide evidence of differential skill retention. This cautious interpretation is particularly important because procedural-skill performance after simulation may vary according to the procedure, assessment method, and reassessment interval [20].

The findings should also be interpreted in relation to the educational conditions compared. UIS-LP combined faculty-led instruction, lumbar sonoanatomy training, preprocedural ultrasound localization, supervised simulated LP practice, repeated practice, feedback, debriefing, and management of a standardized simulated intraprocedural event. In contrast, SDL consisted of independent access to standardized educational materials and was not matched for instructor contact or hands-on procedural exposure. The observed between-group differences therefore characterize the multicomponent UIS-LP program as delivered relative to SDL and cannot determine the contribution of ultrasound, simulation practice, instructor interaction, feedback, or any other individual component. Furthermore, because procedural performance was assessed exclusively in simulation, the findings do not establish patient-based procedural competence, transfer to clinical LP performance, or improved patient safety.

### 4.2. Ultrasound-Integrated Simulation and Multidimensional Procedural Learning

The secondary outcomes provide a multidimensional view of performance following the two educational conditions. Procedural knowledge represented a cognitive outcome, ultrasound sonoanatomy identification assessed image-based anatomical recognition, self-confidence reflected learners’ self-reported perceptions, and simulated vasovagal reaction management assessed performance within a predefined procedural event. Although all four immediate secondary comparisons remained below the post hoc Bonferroni threshold of α = 0.0125, these outcomes remained exploratory because the multiplicity analysis was post hoc and the assessments differed in baseline measurement and construct.

Structured practice, repeated performance, feedback, and assessment are established features of simulation-based procedural education [5,23,24]. Previous LP studies have also reported higher post-training procedural-performance scores following simulation-based mastery learning and deliberate practice [3,6]. UIS-LP incorporated these educational elements together with lumbar sonoanatomy instruction, preprocedural ultrasound localization, supervised simulated LP practice, and management of a standardized simulated intraprocedural event. Outcomes across these domains should therefore be interpreted in relation to the multicomponent educational condition rather than attributed to any individual component.

Ultrasound was used for lumbar sonoanatomy identification and preprocedural localization rather than real-time visualization or guidance of needle advancement. Higher post-intervention sonoanatomy identification scores were observed in the UIS-LP group. This finding can be considered within the broader literature describing ultrasound anatomy, hands-on ultrasound education, and ultrasound-related procedural simulation in medical education [12,13,14,15]. However, the sonoanatomy assessment was administered only after the educational conditions, was image-based, and closely corresponded to UIS-LP content. It did not assess probe handling, image acquisition or optimization, real-time needle guidance, or patient-based ultrasound-assisted LP. The between-group difference therefore represents performance on the specified sonoanatomy assessment and does not establish within-participant change, comprehensive practical ultrasound proficiency, an independent contribution of ultrasound, or transfer to patient-based performance.

Procedural knowledge requires separate interpretation because the groups differed on this measure at baseline. The post hoc ANCOVA adjusting for baseline procedural knowledge yielded an adjusted between-group mean difference of 3.17 points (95% CI, 2.46–3.89; *p* < 0.001), which was used as the principal inferential comparison for this exploratory secondary outcome. Although this comparison remained below the post hoc Bonferroni threshold, both its exploratory status and the post hoc nature of the adjustment should remain explicit. Baseline procedural knowledge was also included in a separate post hoc sensitivity analysis of the primary OSCE outcome, which showed an adjusted mean difference of 8.29 points (95% CI, 6.32–10.27; *p* < 0.001). Because procedural knowledge and directly observed procedural performance represent different constructs, and because no baseline OSCE was administered, this sensitivity analysis cannot establish baseline comparability in procedural performance.

Self-confidence also represents a construct distinct from directly observed performance. Higher immediate post-intervention self-confidence scores were observed in the UIS-LP group; however, these scores reflect learners’ perceptions of their capabilities rather than demonstrated procedural competence. The study did not assess the relationship between self-confidence and subsequent supervised patient-based LP performance. Accordingly, the self-confidence findings should not be interpreted as evidence of clinical competence or transfer to clinical practice.

Simulated vasovagal reaction management constituted a separate performance domain and was not part of the 14-item LP OSCE. The standardized scenario assessed predefined responses to one simulated intraprocedural event, including recognition, interruption of the procedure when appropriate, supportive measures, communication, assistance-seeking, reassessment, and escalation. Vasovagal reactions and their management are described in the clinical literature [16,17], while broader simulation and crisis-resource-management literature has addressed structured practice involving communication, recognition, teamwork, and responses to evolving clinical situations [18,19]. Nevertheless, the assessment was administered only after the educational conditions and was closely aligned with UIS-LP content. The observed between-group difference therefore applies to performance within this standardized scenario and does not establish comprehensive LP complication-management capability or patient-based clinical performance.

The post-intervention-only design of the sonoanatomy and vasovagal assessments further limits interpretation. Neither assessment permits estimation of within-participant change, and both corresponded closely to tasks incorporated into UIS-LP. Assessment using unfamiliar ultrasound images, different anatomical presentations, or procedural events not encountered during training would be required to examine performance beyond the specifically trained and assessed tasks.

The 2–4-week reassessment provides information about procedural performance beyond the immediate assessment but does not demonstrate differential skill retention. Although OSCE scores were higher in the UIS-LP group at reassessment, the group × time interaction was not statistically significant (β = 0.38 points; 95% CI, −1.67 to 2.44; *p* = 0.714). The same OSCE framework was used at both post-intervention assessments, so testing or recall effects cannot be excluded. These considerations are relevant because maintenance of procedural performance after simulation may vary according to the procedure, assessment method, and reassessment interval [20].

Taken together, the secondary outcomes document between-group differences across several specified educational assessments but do not identify the mechanism underlying those differences. UIS-LP combined instructor-led teaching, preprocedural ultrasound exposure, supervised hands-on simulation, repeated practice, feedback, debriefing, and simulated event management, whereas SDL consisted of independent educational materials without matched instructor contact or hands-on procedural exposure. The findings therefore characterize the multicomponent UIS-LP program as delivered relative to SDL and should not be interpreted as evidence of an isolated ultrasound contribution, transfer to patient-based LP performance, or broader clinical competence.

### 4.3. Ultrasound-Integrated Lumbar Puncture Training, Patient Safety, and Complication Management

Point-of-care ultrasonography can provide anatomical information that complements physical examination during procedural planning [25]. In neuraxial procedures, preprocedural ultrasound has been used to identify vertebral levels, relevant lumbar structures, interlaminar spaces, and potential puncture locations [9,10,11,26]. In the present study, ultrasound was restricted to lumbar sonoanatomy identification and preprocedural localization and was not used for real-time visualization or guidance of needle advancement. The higher post-intervention sonoanatomy identification scores observed in the UIS-LP group should therefore be interpreted within this specific educational application.

The ultrasound assessment evaluated recognition and interpretation of standardized lumbar ultrasound images. It did not assess probe handling, image acquisition or optimization, real-time needle guidance, or ultrasound-assisted LP performance in patients. Moreover, the assessment was administered only after the educational conditions and was closely aligned with UIS-LP content. The observed between-group difference therefore represents performance on the specified image-based assessment and does not establish within-participant change, comprehensive practical ultrasound proficiency, an independent contribution of ultrasound, or transfer to patient-based procedures.

LP performance involves considerations beyond anatomical localization, including patient positioning, aseptic technique, procedural sequencing, recognition of contraindications, and responses to procedural concerns [1,2]. UIS-LP addressed these elements within supervised simulation, repeated practice, feedback, and debriefing, consistent with established approaches to simulation-based procedural education [5,23,24]. Because these elements were incorporated within the same educational condition, the study cannot determine the individual contribution of ultrasound, supervised practice, feedback, or any other component to the observed between-group difference in procedural performance.

Management of a standardized simulated vasovagal reaction provided a separate assessment of responses to one predefined intraprocedural event. Vasovagal reactions can include nausea, diaphoresis, hypotension, bradycardia, and transient loss of consciousness [16,17]. The study scenario assessed recognition, interruption of the procedure when appropriate, positioning and supportive measures, communication and reassurance, assistance-seeking, reassessment, and escalation when indicated. Broader simulation literature has addressed crisis-resource-management approaches involving recognition, communication, teamwork, and responses to evolving clinical situations [18,19]. These references provide educational context for the simulated event but do not constitute evidence specific to vasovagal management during LP.

The vasovagal findings should therefore remain within the boundaries of the assessment. The scenario represented one standardized event and was evaluated separately from the 14-item LP procedural-performance OSCE. It was not intended to represent the relative frequency or severity of vasovagal reactions among LP-related complications and was not a comprehensive assessment of complication management. Although patient selection, contraindications, and procedural safety were addressed elsewhere in the educational content, other LP-related complications were not evaluated through separate standardized simulation scenarios. The observed between-group difference consequently represents performance on the specified vasovagal assessment rather than broader complication-management capability.

This distinction is particularly important when considering patient safety. Simulation provides a controlled educational environment in which procedural tasks and responses to simulated clinical events can be practiced and assessed [5,23,24]. However, the present study did not assess patient-based LP success, first-attempt success, number of needle attempts or passes, need for supervisor assistance, patient discomfort, or procedure-related complications. The simulation-based findings therefore do not establish improved patient safety, independent clinical competence, or transfer to patient-based LP performance.

The sonoanatomy and vasovagal assessments also provide limited information regarding performance beyond the specifically trained and assessed tasks because both were post-intervention measures closely aligned with UIS-LP content. Assessment using unfamiliar ultrasound images, different anatomical presentations, or procedural events not encountered during training would be required to examine performance under less closely aligned conditions. Whether the observed between-group differences in simulation are also present during clinical LP performance requires direct assessment during appropriately supervised patient-based procedures.

### 4.4. Comparison with Previous Simulation Studies

The present findings can be considered alongside previous research on simulation-based LP education. Earlier studies have reported higher post-training procedural-performance scores following simulation-based mastery learning and deliberate practice [3,6]. Simulation-based education has also been evaluated in other procedural and acute-care contexts, including paracentesis training and medical-student education involving simulated severe-sepsis management [27,28]. Although these studies differ in procedure, learner population, educational design, and assessment strategy, they provide broader context for structured simulation in procedural education.

Simulation-based procedural education has been delivered through diverse modalities across specialties. Regional-anesthesia literature describes the use of simulation for procedural and ultrasound-related education [29]. Specific approaches include low-cost, low-fidelity training for ultrasound-guided nerve blocks [30], virtual-reality training for ultrasound-guided needling [31], comparison of cadaveric and phantom models for teaching ultrasound-guided regional anesthesia [32], and cognitive simulation for procedural-skills learning among medical students [33]. These studies illustrate different approaches to procedural simulation but are not directly equivalent to the multicomponent UIS-LP program evaluated in the present study.

The present study assessed several distinct educational outcomes within the same randomized comparison. In addition to immediate simulation-based LP procedural performance, assessments included procedural knowledge, ultrasound sonoanatomy identification, self-confidence, simulated vasovagal reaction management, and procedural performance at 2–4 weeks. These measures represent different constructs and should not be interpreted interchangeably. Procedural knowledge required baseline adjustment because of the observed baseline difference, whereas sonoanatomy identification and vasovagal reaction management were assessed only after the educational conditions and therefore do not provide estimates of within-participant change.

The ultrasound component should also be distinguished from studies specifically examining ultrasound-guided procedural training. Previous literature describes ultrasound and simulation-based education across medical and regional-anesthesia settings [12,13,14,15,29,30,31,32]. Separately, ultrasound has been described as providing anatomical information relevant to procedural planning and neuraxial localization [25,26]. In the present study, ultrasound was restricted to lumbar sonoanatomy instruction and preprocedural localization and was not used for real-time guidance of needle advancement. Ultrasound exposure was also embedded within faculty-led instruction, supervised simulation, repeated practice, feedback, and debriefing. The higher post-intervention sonoanatomy scores therefore represent a between-group difference on the specified image-based assessment and do not establish an independent contribution of ultrasound or practical ultrasound-guided LP proficiency.

The standardized simulated intraprocedural event can be considered within a separate body of simulation literature. Simulation-based training has been evaluated for healthcare-team human-factor skills, including communication and teamwork [34]. Serious-game approaches have been studied in cardiopulmonary resuscitation education [35], and interprofessional simulation using Emergency Crisis Resource Management principles has been evaluated in emergency-team training [36]. These studies provide broader educational context for incorporating communication, assistance-seeking, teamwork, and responses to evolving simulated situations. They do not, however, provide direct evidence regarding LP-specific complication management or vasovagal reactions during LP. The present finding should therefore remain interpreted as performance within one predefined simulated vasovagal scenario.

The 2–4-week reassessment extends measurement beyond the immediate post-intervention assessment but should not be interpreted as evidence of differential skill retention. A between-group difference in OSCE procedural performance was observed at reassessment, whereas the group × time interaction was not statistically significant. A systematic review of procedural-skill retention after simulation indicates that subsequent performance may vary according to the procedure, assessment method, and reassessment interval [20]. In the present study, the same OSCE framework was used at both post-intervention assessments, and testing or recall effects cannot be excluded.

Finally, differences in educational exposure are important when comparing the present study with previous simulation studies. UIS-LP included instructor-led teaching, preprocedural ultrasound exposure, supervised hands-on simulated LP practice, repeated practice, feedback, and debriefing, whereas SDL consisted of independent access to standardized educational materials. The conditions were not matched for instructor contact or hands-on procedural exposure, and participant-level training exposure was not quantified. Consequently, the observed between-group differences characterize the multicomponent UIS-LP program as delivered relative to SDL and do not determine the contribution of ultrasound, faculty interaction, hands-on simulation, feedback, or any other individual component.

### 4.5. Educational Implications

The findings may inform the organization of undergraduate lumbar puncture (LP) education, but their implications should remain within the educational conditions and outcomes evaluated. LP training encompasses anatomical localization, patient positioning, aseptic technique, procedural sequencing, technical performance, communication, and responses to procedural concerns. The higher immediate Objective Structured Clinical Examination (OSCE) scores observed in the UIS-LP group represent higher performance on the standardized simulation-based assessment relative to SDL and should not be interpreted as evidence of readiness for independent patient-based LP or broader clinical competence.

Within undergraduate curricula, a multicomponent approach such as UIS-LP could complement existing procedural instruction and appropriately supervised clinical exposure. Repeated practice, structured feedback, and performance assessment are established elements of simulation-based procedural education [5,23,24]. Simulation-based approaches have also been evaluated across a range of procedural and acute-care contexts using different instructional modalities [27,28,29,30,31,32,33]. These studies provide broader educational context rather than direct evidence for UIS-LP. The curricular role of such a program would depend on learner experience, intended learning outcomes, available faculty and simulation resources, instructional time, and opportunities for subsequent supervised clinical practice.

Technology-enhanced and alternative instructional approaches provide additional context for curriculum design. Virtual-reality simulation has been evaluated among nursing students [37], while flipped-classroom approaches have been examined across undergraduate health-professions education [38]. Different teaching modes have also been compared in relation to medical-student assessment performance [39], and a simulation-based clinical research curriculum has been evaluated among undergraduate medical students [40]. Although these studies address educational settings and outcomes that differ from LP procedural training, they illustrate the range of instructional formats available for consideration when designing undergraduate educational programs.

The incorporation of preprocedural ultrasound localization and a standardized simulated intraprocedural event broadened the tasks included within UIS-LP. Literature on neuraxial ultrasound and ultrasound education provides context for lumbar anatomical identification and preprocedural localization [9,10,11,12,13,14,15,25,26], whereas clinical vasovagal literature and broader crisis-resource-management simulation literature provide context for the standardized simulated event [16,17,18,19,34,35,36]. In the present study, these elements were delivered together with faculty instruction, supervised hands-on simulation, repeated practice, feedback, and debriefing. The study therefore does not permit their individual contributions to be separated from the multicomponent program as delivered.

The secondary outcomes may identify domains relevant to subsequent instruction or supervised practice but should not be interpreted as evidence of clinical transfer. Procedural knowledge required baseline adjustment because of the observed between-group difference, while ultrasound sonoanatomy identification and vasovagal reaction management were post-intervention assessments closely aligned with UIS-LP content. Self-confidence was self-reported and does not substitute for directly observed procedural performance. These outcomes therefore provide complementary information about performance on the specified educational assessments rather than evidence of patient-based LP capability.

An important curricular distinction is that between performance demonstrated in simulation and performance during supervised patient care. Simulation-based education for other invasive procedures, including central venous catheterization, has been evaluated using outcomes extending beyond immediate learner assessment [41]. More broadly, simulation provides a controlled educational environment for practicing clinical and procedural tasks [42]. The present study, however, did not assess patient-based LP performance or patient outcomes. Simulation-based OSCE performance should therefore be considered a measure of performance within the study environment rather than evidence that a learner is prepared to perform LP independently in clinical practice.

The educational contrast between UIS-LP and SDL should also guide interpretation. UIS-LP included instructor-led teaching, supervised ultrasound exposure, hands-on simulated LP practice, repeated practice, feedback, and debriefing, whereas SDL consisted of independent access to standardized educational materials without matched instructor contact or hands-on procedural exposure. Consequently, the observed between-group differences cannot determine the contribution of ultrasound, simulation practice, faculty interaction, feedback, or any other individual component. Future comparisons using attention-matched, time-matched, or hands-on comparator conditions could provide more specific information about the contribution of individual program components.

The 2–4-week reassessment should not be used to define a refresher-training interval. Although a between-group difference in OSCE procedural performance was observed at reassessment, the group × time interaction was not statistically significant. The study therefore provides information about procedural performance at 2–4 weeks but does not demonstrate differential skill retention or identify an optimal interval for retraining. Longer follow-up with prospectively specified reassessment intervals and approaches that reduce potential testing or recall effects would provide a stronger basis for examining maintenance of procedural performance [20].

UIS-LP may therefore warrant further evaluation as a preparatory component of undergraduate procedural education used alongside, rather than as a substitute for, appropriately supervised clinical training. Future studies should directly assess whether between-group differences observed in simulation are also present during supervised patient-based LP and should separately examine feasibility, resource requirements, educational exposure, and the contribution of individual program components.

### 4.6. Study Strengths and Limitations

This study has several strengths. The randomized parallel-group design incorporated allocation concealment and blinded outcome assessment. The prespecified primary outcome was evaluated using a standardized 14-item simulation-based lumbar puncture (LP) procedural-performance Objective Structured Clinical Examination (OSCE) with item-specific scoring criteria and two faculty assessors blinded to group allocation. The assessment instruments underwent expert review and pilot testing. In addition, all 78 randomized participants completed the immediate assessments and the 2–4-week OSCE reassessment, providing complete procedural-performance follow-up.

Several limitations should also be considered. First, the study was conducted at a single institution among clinical-year medical students. Generalizability to settings with different curricula, learner populations, faculty expertise, or simulation and ultrasound resources is therefore uncertain.

Second, baseline differences were present despite randomization. Procedural knowledge was higher in the UIS-LP group, whereas participants allocated to SDL were older. More importantly, no baseline OSCE was administered, preventing direct assessment of baseline comparability in procedural performance. The prespecified unadjusted immediate OSCE comparison therefore remained the primary analysis. The post hoc ANCOVA adjusting for baseline procedural knowledge was conducted as a sensitivity analysis but cannot substitute for a baseline procedural-performance measure because cognitive knowledge and directly observed procedural performance represent different constructs. For procedural knowledge itself, the baseline-adjusted ANCOVA estimate was used as the principal inferential comparison; however, this analysis was post hoc and the outcome remained exploratory.

Third, blinding of participants and instructors was not feasible because the educational conditions were readily distinguishable. Differences in expectations, engagement, motivation, or instructor interaction could therefore have influenced performance. Participants were instructed not to share study-specific educational materials or assessment content between groups, but adherence to this instruction and potential cross-group contamination were not formally assessed. Additional LP- or ultrasound-related educational exposure outside the assigned conditions was also not systematically recorded.

Fourth, the educational exposures differed substantially and were incompletely quantified. UIS-LP consisted of a 2 h instructor-led session incorporating supervised ultrasound exposure and simulated LP practice, whereas SDL involved 2 weeks of independent access to standardized educational materials. Individual SDL study duration and electronic resource use were not recorded, and participant-level UIS-LP hands-on practice time, number of ultrasound scans, and number of simulated LP attempts were not documented. Differences in instructor contact, hands-on exposure, and practice intensity therefore cannot be separated from the overall comparison between the educational conditions.

Fifth, UIS-LP was evaluated as a multicomponent educational program. Faculty-led instruction, lumbar sonoanatomy training, preprocedural ultrasound localization, supervised simulated LP practice, repeated practice, feedback, debriefing, and simulated vasovagal reaction management were delivered together. The study was not designed to estimate the contribution of individual components; consequently, the observed between-group differences cannot be attributed specifically to ultrasound or any other individual component of UIS-LP.

Sixth, some secondary assessments were particularly susceptible to content alignment. Ultrasound sonoanatomy identification and simulated vasovagal reaction management were assessed only after the educational conditions and corresponded closely to UIS-LP content. These assessments therefore characterize post-intervention performance on the specified tasks rather than within-participant change or performance beyond the trained content. The vasovagal scenario represented only one predefined intraprocedural event and should not be interpreted as a comprehensive assessment of LP complication management or patient safety.

Seventh, the secondary analyses remained exploratory. Although all four immediate secondary comparisons were below the post hoc Bonferroni threshold of α = 0.0125, this multiplicity sensitivity analysis was not prespecified. The baseline-adjusted analysis of procedural knowledge and the ANCOVA sensitivity analysis of the primary OSCE outcome were also post hoc. These analyses should therefore remain clearly distinguished from the prespecified primary OSCE analysis.

Eighth, all directly observed procedural-performance outcomes were obtained in simulation. The study did not evaluate patient-based LP performance, procedural success, first-attempt success, number of needle attempts or passes, need for supervisor assistance, patient discomfort, or procedure-related complications. The findings therefore do not establish patient-based procedural competence, transfer to clinical LP performance, or improved patient safety.

Finally, follow-up was limited to 2–4 weeks. Although a between-group difference in OSCE procedural performance was observed at reassessment, the group × time interaction was not statistically significant; therefore, the study does not demonstrate differential skill retention or identify an optimal interval for retraining. The same OSCE framework was used at both post-intervention assessments, so testing or recall effects cannot be excluded. Furthermore, although all participants completed reassessment within the prespecified 2–4-week window, exact participant-level reassessment intervals were not retained, preventing examination of whether variation in timing within this window was associated with performance.

Taken together, the randomized design, allocation concealment, blinded OSCE assessment, standardized primary outcome, and complete procedural-performance follow-up strengthen the internal evaluation of the educational comparison. These strengths should be considered alongside the absence of a baseline OSCE, observed baseline differences, unequal and incompletely quantified educational exposure, potential unmeasured contamination, post hoc analyses, inability to isolate individual UIS-LP components, simulation-only assessment, and limited follow-up. The findings are therefore most appropriately interpreted as between-group differences in performance on the specified educational assessments under the conditions evaluated.

### 4.7. Future Research

Future multicenter studies should examine whether the observed between-group differences are reproducible across institutions, curricula, learner populations, and simulation environments. Baseline procedural performance should be assessed using the same or a comparable OSCE to permit direct evaluation of baseline procedural ability and subsequent change. Where appropriate, prespecified blocked or stratified randomization based on relevant learner characteristics could also be considered in studies with modest sample sizes to reduce the likelihood of important baseline imbalance.

A major priority is to determine whether between-group differences observed in simulation are also present during appropriately supervised patient-based LP performance. Future studies could prospectively document procedures observed, assisted, and performed; use of preprocedural ultrasound localization; number of needle attempts or passes; first-attempt and overall procedural success; and level of supervisor assistance. Standardized supervisor-assessed measures could complement procedural logbooks and permit direct comparison of performance across simulated and supervised clinical settings. Self-confidence should be evaluated alongside, rather than used as a surrogate for, directly observed procedural performance [5,23,24].

Performance beyond tasks closely aligned with UIS-LP should also be examined. Future assessments could incorporate unfamiliar ultrasound images, different anatomical presentations, patient-selection and contraindication decisions, and procedural events not encountered during training. Ultrasound-related assessments could distinguish image recognition and interpretation from probe handling, image acquisition, image optimization, and procedural application [12,13,14,15,25,26]. Such designs would provide information about performance under conditions less closely aligned with the original training and assessment tasks without presuming transfer to clinical practice.

The contribution of individual UIS-LP components also requires separate investigation. Attention-matched or time-matched comparators, factorial designs, or other component-focused approaches could permit more specific evaluation of preprocedural ultrasound localization, supervised simulation, repeated practice, feedback, and debriefing. Future studies should prospectively quantify instructor contact, hands-on training time, number of ultrasound scans and simulated LP attempts, achievement of predefined performance criteria, SDL engagement, and additional LP- or ultrasound-related educational exposure. Because SDL approaches and their assessment vary across health-professions education, learner engagement with assigned SDL materials should also be characterized prospectively [21,22].

Longer follow-up is needed to characterize procedural performance beyond the 2–4-week period. Reassessment intervals should be specified prospectively, and longitudinal studies could incorporate novel or less closely aligned tasks to reduce potential testing or recall effects. Where feasible, simulation-based reassessment could be complemented by procedural logbooks and standardized assessments during supervised clinical practice. Procedural-skill performance after simulation may vary according to the procedure, assessment approach, and reassessment interval [20]. Because the group × time interaction in the present study was not statistically significant, the current findings do not demonstrate differential skill retention or provide a basis for specifying a refresher-training interval.

Future comparative studies could also evaluate alternative approaches to procedural education while carefully controlling educational exposure. Regional-anesthesia and procedural-education literature describes simulation using conventional models, low-cost task trainers, virtual-reality environments, and cognitive simulation [29,30,31,32,33], while virtual-reality simulation has also been evaluated in other health-professions learners [37]. Alternative instructional formats, including flipped-classroom approaches and other teaching modes, have been examined in undergraduate health-professions and medical education [38,39,40]. These studies provide potential methodological comparators rather than direct evidence for LP education. Future comparisons should clearly specify instructional content, faculty contact, hands-on practice, learner engagement, and comparator exposure.

The event-management component could likewise be examined using additional standardized scenarios. Future assessments could extend beyond the vasovagal reaction to procedural events not encountered during the original training and could evaluate recognition, communication, assistance-seeking, reassessment, escalation, and, where appropriate, team-based responses. Broader simulation literature has examined human-factor skills [34], serious-game approaches to resuscitation education [35], interprofessional Emergency Crisis Resource Management simulation [36], and technology-enhanced simulation [37]. These approaches provide potential models for future study design but should not be assumed to apply directly to LP-specific event management.

Future studies should also distinguish educational assessment outcomes from subsequent clinical outcomes. Simulation-based education for central venous catheterization has been examined using outcomes extending beyond learner assessment scores [41], while broader literature describes simulation as a controlled environment for experiential clinical learning [42]. For UIS-LP, subsequent research should directly assess whether differences observed in simulation are also present during supervised patient-based LP rather than assuming transfer between settings. Patient-based outcomes, if examined, should be prospectively defined and assessed separately from simulation-based performance.

Analytical approaches should also be prespecified. For outcomes measured both before and after the educational conditions, appropriate baseline-adjusted analyses should be defined before data analysis, together with prespecified procedures for addressing multiplicity across secondary outcomes. In particular, inclusion of a baseline procedural-performance assessment would permit direct adjustment for baseline OSCE performance rather than using procedural knowledge, a distinct cognitive construct, as an indirect covariate.

Finally, implementation research could examine faculty and simulation-technician workload, instructor standardization, equipment requirements, learner throughput, scheduling, costs, and performance benchmarks. Evaluation of these factors alongside simulation-based performance and, where feasible, appropriately supervised patient-based assessment would provide information about the feasibility, scalability, and curricular integration of UIS-LP across undergraduate medical education settings. Such studies should continue to distinguish evaluation of the multicomponent UIS-LP program as delivered from evaluation of ultrasound or any other individual component.

## 5. Conclusions

Among clinical-year medical students, immediate simulation-based lumbar puncture (LP) procedural-performance scores were higher in the UIS-LP group than in the SDL group. Between-group differences were also observed across the exploratory secondary outcomes. For procedural knowledge, the baseline-adjusted estimate was used as the principal inferential comparison because of the observed baseline difference.

At the 2–4-week reassessment, OSCE procedural-performance scores were higher in the UIS-LP group than in the SDL group; however, the group × time interaction was not statistically significant. The reassessment therefore reflects a between-group difference in procedural performance at 2–4 weeks rather than evidence of differential skill retention.

These findings characterize the multicomponent UIS-LP program as delivered relative to SDL and cannot be attributed specifically to ultrasound or any other individual component. Because procedural performance was assessed in simulation, the findings do not establish patient-based procedural competence, transfer to clinical LP performance, or improved patient safety.

## Figures and Tables

**Figure 1:**
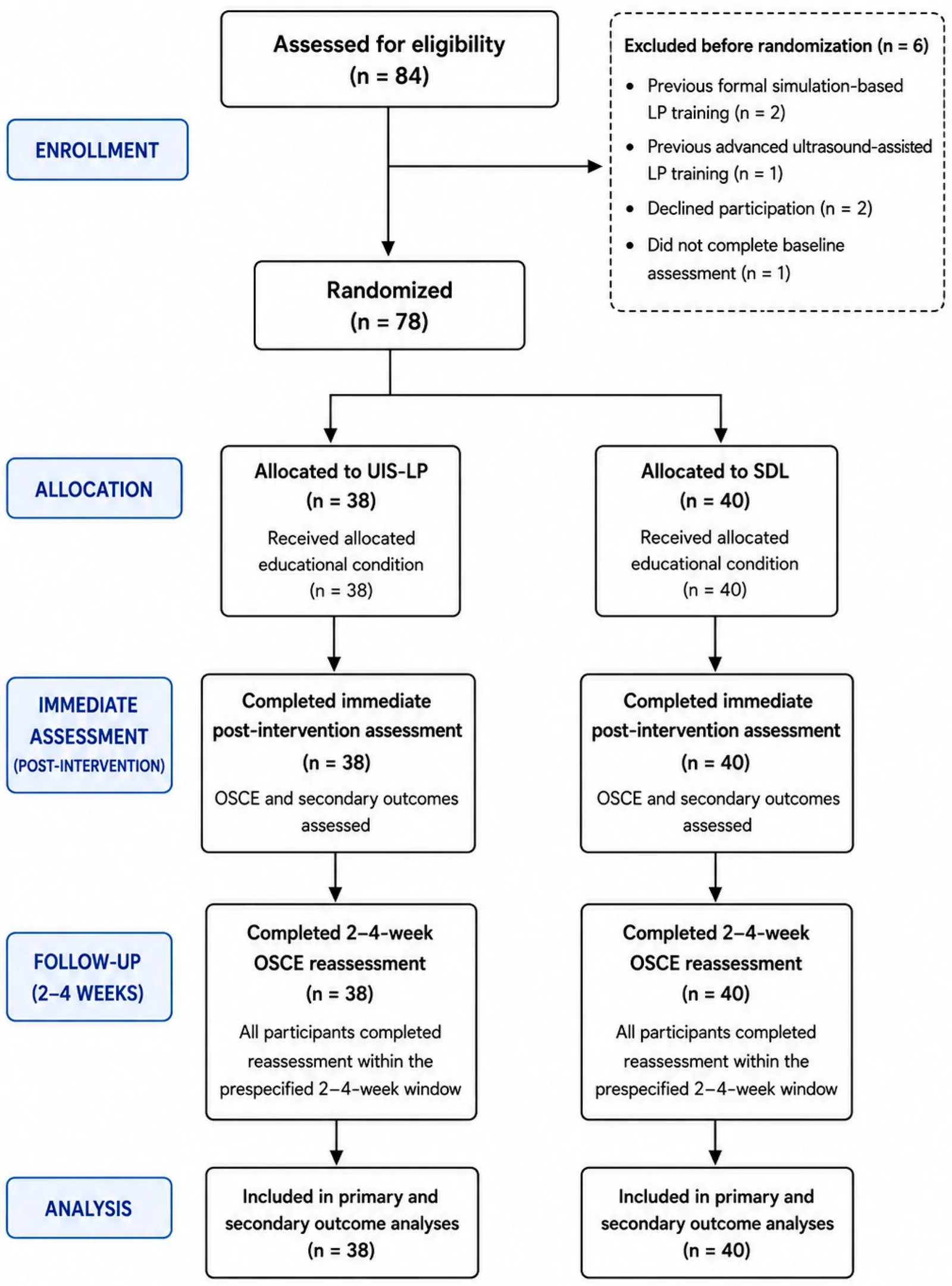
Participant flow through the randomized controlled educational trial. Of 84 clinical-year medical students assessed for eligibility, 78 were randomized to UIS-LP (*n* = 38) or SDL (*n* = 40). All randomized participants completed the allocated educational condition, immediate post-intervention assessments, and OSCE reassessment within the prespecified 2–4-week window. LP, lumbar puncture; OSCE, Objective Structured Clinical Examination; SDL, self-directed learning; UIS-LP, ultrasound-integrated simulation-based lumbar puncture.

**Figure 2:**
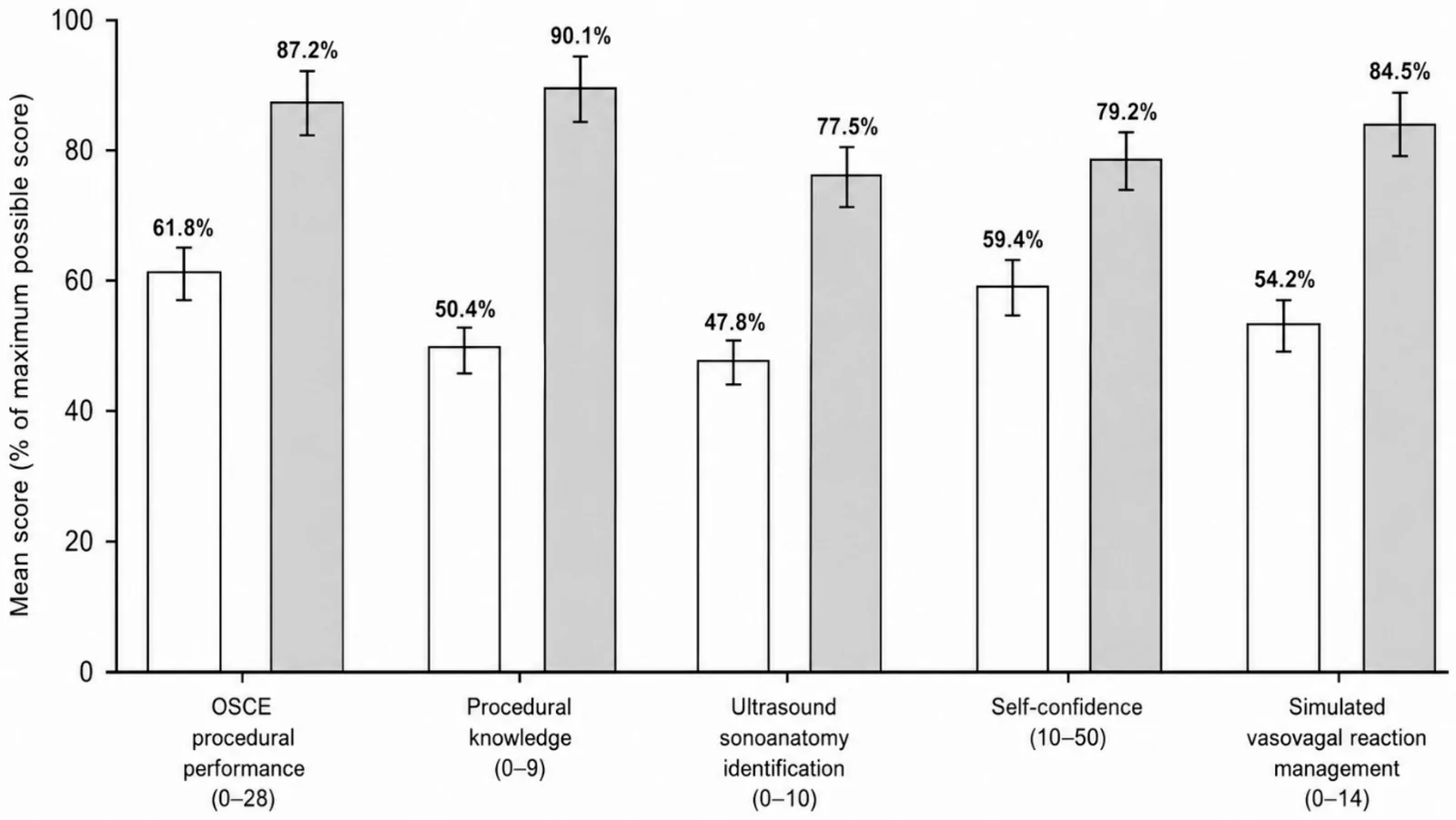
**Normalized immediate post-intervention outcomes.** Mean scores are expressed as percentages of the maximum possible score for each assessment to facilitate descriptive comparison across measures with different score ranges. Values shown are the observed, unadjusted post-intervention means for the self-directed learning (SDL; *n* = 40) and ultrasound-integrated simulation-based lumbar puncture (UIS-LP; *n* = 38) groups. Normalization was used for descriptive graphical presentation only; all inferential analyses were conducted using the original, non-normalized scores. For procedural knowledge, the baseline-adjusted ANCOVA estimate reported in Section 3 and Table 5 was used for inference and is not represented in this figure. OSCE, Objective Structured Clinical Examination; SDL, self-directed learning; UIS-LP, ultrasound-integrated simulation-based lumbar puncture.

**Table 1:** Baseline Demographic and Educational Characteristics of Participants.

Variable	SDL (*n* = 40)	UIS-LP (*n* = 38)	Total (*n* = 78)	Standardized Difference
**Age, years, mean ± SD**	22.55 ± 1.18	21.74 ± 0.75	22.15 ± 1.06	−0.81
**Sex, *n* (%)**				
Female	24 (60.0)	22 (57.9)	46 (59.0)	−0.04
Male	16 (40.0)	16 (42.1)	32 (41.0)	—
**Academic year, *n* (%)**				0.48 ^a^
Fourth year	12 (30.0)	5 (13.2)	17 (21.8)	—
Fifth year	20 (50.0)	27 (71.1)	47 (60.3)	—
Sixth year	8 (20.0)	6 (15.8)	14 (17.9)	—
**Previous LP exposure, *n* (%)**				
Yes	12 (30.0)	8 (21.1)	20 (25.6)	−0.21
No	28 (70.0)	30 (78.9)	58 (74.4)	—

**Note:** Data are presented as mean ± SD or n (%). Baseline characteristics were summarized descriptively without hypothesis testing. Standardized differences are expressed as UIS-LP − SDL. For continuous variables, standardized mean differences were calculated using the pooled SD; for binary variables, standardized differences were calculated using pooled proportional variance. For binary characteristics, the standardized difference is reported once rather than separately for complementary categories. LP, lumbar puncture; SD, standard deviation; SDL, self-directed learning; UIS-LP, ultrasound-integrated simulation-based lumbar puncture. ^a^ Overall standardized difference for the three-category academic-year distribution.

**Table 2:** Baseline Procedural Knowledge and Self-Confidence Scores.

Measure	SDL (*n* = 40)	UIS-LP (*n* = 38)	Standardized Difference
Procedural knowledge score (0–9), mean ± SD	3.15 ± 1.83	6.08 ± 1.67	1.67
Median (range)	3.0 (1–8)	6.5 (3–9)	—
Percentage of maximum score, %	35.0	67.6	—
Self-confidence score (10–50), mean ± SD	27.0 ± 9.9	25.6 ± 6.0	−0.17

**Note:** Data are presented as mean ± SD, median (range), or percentage, as appropriate. Baseline measures were summarized descriptively without hypothesis testing. Standardized differences are expressed as UIS-LP − SDL and were calculated using the pooled SD. Higher scores indicate greater procedural knowledge or self-reported confidence. A substantial observed baseline difference was present in procedural knowledge (standardized difference, 1.67). Accordingly, the immediate post-intervention procedural-knowledge comparison was adjusted for baseline procedural knowledge using post hoc ANCOVA, with the baseline-adjusted estimate used as the principal inferential comparison for this exploratory secondary outcome. Baseline OSCE procedural performance was not assessed; therefore, procedural knowledge should not be interpreted as a baseline measure or surrogate of directly observed LP procedural performance. ANCOVA, analysis of covariance; LP, lumbar puncture; OSCE, Objective Structured Clinical Examination; SD, standard deviation; SDL, self-directed learning; UIS-LP, ultrasound-integrated simulation-based lumbar puncture.

**Table 3:** Baseline Self-Confidence Scores by Domain.

Self-Confidence Domain	Corresponding S2 Items	SDL (*n* = 40)	UIS-LP (*n* = 38)
Anatomical landmark identification	Items 1–2	2.55 ± 1.15	2.79 ± 0.66
Patient positioning	Items 3–4	2.55 ± 1.22	2.71 ± 0.57
Procedural steps	Items 5–6	2.70 ± 1.09	2.50 ± 0.76
Contraindications and procedural safety	Items 7–8	2.65 ± 1.37	2.39 ± 0.97
Aseptic technique	Items 9–10	3.05 ± 1.36	2.42 ± 0.89

**Note:** Data are presented as mean ± SD. Each domain comprises two items from the 10-item self-confidence questionnaire (Supplementary Table S2), rated on a five-point Likert scale. Domain scores represent the mean of the corresponding items, with higher scores indicating greater self-reported confidence. Domain-level results are descriptive; no between-group hypothesis tests were performed. The total baseline self-confidence score (range, 10–50) is reported in Table 2. SD, standard deviation; SDL, self-directed learning; UIS-LP, ultrasound-integrated simulation-based lumbar puncture.

**Table 4:** Immediate Post-Intervention Outcomes.

Outcome	SDL (*n* = 40), Mean ± SD	UIS-LP (*n* = 38), Mean ± SD	Mean Difference (UIS-LP − SDL)	95% CI	Cohen’s *d*	*p* Value
OSCE procedural performance (0–28)	17.29 ± 4.64	24.42 ± 2.13	7.13	5.49–8.77	1.96	<0.001
Procedural knowledge (0–9) ^a^	4.54 ± 1.68	8.11 ± 0.68	3.57	2.98–4.15	2.76	<0.001
Ultrasound sonoanatomy identification (0–10)	4.78 ± 1.81	7.75 ± 1.05	2.97	2.30–3.64	1.99	<0.001
Self-confidence (10–50)	29.71 ± 7.24	39.60 ± 4.40	9.89	7.17–12.61	1.64	<0.001
Simulated vasovagal reaction management (0–14)	7.59 ± 2.26	11.83 ± 1.41	4.24	3.38–5.09	2.24	<0.001

**Note:** Data are observed, unadjusted immediate post-intervention scores and are presented as mean ± SD. Mean differences are expressed as UIS-LP − SDL with 95% confidence intervals (CIs). Cohen’s *d* was calculated using the pooled SD. The OSCE procedural-performance comparison was the prespecified primary analysis. The remaining outcomes were exploratory secondary outcomes. ^a^ Because of the observed baseline difference in procedural knowledge, the baseline-adjusted ANCOVA estimate presented in Table 5 was used as the principal inferential comparison for procedural knowledge. The unadjusted post-intervention estimate shown here is retained as a descriptive supporting result. **Abbreviations:** CI, confidence interval; OSCE, Objective Structured Clinical Examination; SD, standard deviation; SDL, self-directed learning; UIS-LP, ultrasound-integrated simulation-based lumbar puncture.

**Table 5:** Baseline-Adjusted ANCOVA Analyses of Immediate Post-Intervention Outcomes.

Outcome	SDL Adjusted Mean	UIS-LP Adjusted Mean	Adjusted Mean Difference (UIS-LP − SDL)	95% CI	*p* Value
OSCE procedural performance (0–28) ^a^	16.73	25.02	8.29	6.32–10.27	<0.001
Procedural knowledge (0–9) ^b^	4.73	7.90	3.17	2.46–3.89	<0.001

**Note:** Adjusted means and between-group differences were estimated using post hoc ANCOVA, with baseline procedural knowledge as the covariate. Adjusted mean differences are expressed as UIS-LP − SDL with 95% CIs. HC3 heteroscedasticity-consistent robust standard errors were used for statistical inference. ^a^ For the primary OSCE outcome, ANCOVA was conducted as a post hoc sensitivity analysis because baseline OSCE procedural performance was not assessed. The group × baseline procedural knowledge interaction was not statistically significant (*p* = 0.339). The prespecified unadjusted between-group comparison remained the primary analysis. Adjustment for baseline procedural knowledge does not substitute for the unavailable baseline OSCE assessment. ^b^ For procedural knowledge, the post-intervention comparison was adjusted because of the substantial baseline between-group difference. The group × baseline procedural knowledge interaction was not statistically significant (*p* = 0.250). The adjusted mean difference was used as the principal inferential comparison for this exploratory secondary outcome. The unadjusted post-intervention mean difference of 3.57 points (95% CI, 2.98–4.15) is presented in Table 4 as a descriptive supporting result. **Abbreviations:** ANCOVA, analysis of covariance; CI, confidence interval; OSCE, Objective Structured Clinical Examination; SDL, self-directed learning; UIS-LP, ultrasound-integrated simulation-based lumbar puncture.

**Table 6:** Lumbar Puncture Procedural Performance at the 2–4-Week Reassessment.

Outcome	SDL (*n* = 40)	UIS-LP (*n* = 38)	Mean Difference (UIS-LP − SDL)	95% CI	Cohen’s *d*	*p* Value
OSCE procedural performance (0–28), mean ± SD	15.94 ± 3.28	23.45 ± 2.59	7.51	6.17–8.85	2.54	<0.001

Note: Data are presented as mean ± SD. All 78 randomized participants completed the OSCE reassessment within the prespecified 2–4-week window. The mean difference is expressed as UIS-LP − SDL with its 95% CI. The between-group comparison at reassessment used an independent-samples *t* test, and Cohen’s *d* was calculated using the pooled SD. In the longitudinal analysis of OSCE scores across the immediate and 2–4-week post-intervention assessments, the group × time interaction was not statistically significant (β = 0.38 points; 95% CI, −1.67 to 2.44; *p* = 0.714). Accordingly, the result at 2–4 weeks represents a between-group difference in procedural performance at reassessment and should not be interpreted as evidence of differential skill retention or a between-group difference in the pattern of score change over time. Abbreviations: CI, confidence interval; LP, lumbar puncture; OSCE, Objective Structured Clinical Examination; SD, standard deviation; SDL, self-directed learning; UIS-LP, ultrasound-integrated simulation-based lumbar puncture.

## Data Availability

Deidentified participant-level data and the corresponding data dictionary may be made available by the corresponding author upon reasonable request, subject to institutional, ethical, and data-governance requirements. Educational and assessment materials are provided in the manuscript and Supplementary Materials.

## Supplementary Materials

The following supporting information can be downloaded at: https://www.mdpi.com/article/10.3390/jcm15197508/s1.

## Abbreviations

**Abbreviation**	**Full term**
ANCOVA	Analysis of covariance
CI	Confidence interval
CVI	Content Validity Index
HC3	Heteroscedasticity-consistent covariance matrix estimator 3
ICC	Intraclass correlation coefficient
I-CVI	Item-level Content Validity Index
LP	Lumbar puncture
OSCE	Objective Structured Clinical Examination
S-CVI/Ave	Scale-level Content Validity Index using the average method
SD	Standard deviation
SDL	Self-directed learning
UIS-LP	Ultrasound-integrated simulation-based lumbar puncture
