# Supplementary material for: A Multicomponent Simulation-Based Program Incorporating Ultrasound for Lumbar Puncture Training in Medical Students: A Randomized Controlled Educational Trial
Source: J Clin Med. 2026 Sep 27;15(19):7508. doi: 10.3390/jcm15197508 (PMC13665711; doi:10.3390/jcm15197508)
Supplement: Supplementary file 1 [file jcm-15-07508-s001.zip › jcm-4507415-supplementary.pdf]

Supplementary File S1

Assessment Methods and Instruments for Ultrasound-Integrated Simulation-Based Lumbar Puncture Training

Overview

This supplementary file provides detailed information on the assessment instruments, administration procedures, and scoring criteria used in the randomized educational trial comparing a multicomponent ultrasound-integrated simulation-based lumbar puncture (UIS-LP) program with self-directed learning (SDL). It complements the main manuscript by providing the assessment content, response formats, scoring procedures, and behavioral criteria used for the study outcomes.

The assessment framework comprised five domains: procedural knowledge, self-confidence, ultrasound sonoanatomy identification, simulation-based lumbar puncture (LP) procedural performance, and simulated vasovagal reaction management. Procedural knowledge and self-confidence were assessed at baseline and immediately post-intervention. Ultrasound sonoanatomy identification and simulated vasovagal reaction management were assessed only immediately post-intervention. LP procedural performance was assessed immediately post-intervention and reassessed within the prespecified 2–4-week window using the same Objective Structured Clinical Examination (OSCE) framework.

The prespecified primary outcome was immediate post-intervention simulation-based LP procedural performance, assessed using a standardized 14-item OSCE with item-specific 0–2 scoring criteria and a total score ranging from 0 to 28. Procedural knowledge, ultrasound sonoanatomy identification, self-confidence, simulated vasovagal reaction management, and LP procedural performance at the 2–4-week reassessment were secondary outcomes. Baseline OSCE procedural performance was not assessed; therefore, the primary outcome was based on an immediate post-intervention between-group comparison rather than change from baseline.

The assessment instruments were developed or adapted according to the predefined learning objectives and assessment domains. Before participant enrollment, they underwent expert content review addressing item relevance, clarity, suitability for clinical-year medical students, and alignment with the intended domains. The instruments and study procedures were also pilot tested

for clarity, feasibility, administration time, scenario flow, equipment requirements, and application of the scoring framework.

Where retained quantitative records were available, validity and reliability information is reported in the corresponding supplementary sections. For the 10-item self-confidence questionnaire, the documented scale-level Content Validity Index using the average method (S-CVI/Ave) was 0.96, and internal consistency was Cronbach's α = 0.81. Inter-rater reliability for OSCE procedural-performance scoring was ICC = 0.89. Because the original detailed ICC output was unavailable during manuscript revision, the ICC model, agreement definition, confidence interval, and single- versus average-measure specification could not be verified and were not retrospectively reconstructed. Quantitative validity or reliability estimates unavailable in the retained study records were similarly not retrospectively calculated or assigned.

The assessment instruments, item structure, scoring ranges, and administration procedures were established before participant enrollment. The item-specific behavioral anchors presented for the LP OSCE and simulated vasovagal reaction management checklist provide operational descriptions of the original 0–2 scoring framework. These clarifications do not alter the number of items, score ranges, outcome definitions, or participant-level scores used in the analyses.

Assessments were conducted under standardized conditions at the University of Tabuk Simulation Center. At corresponding assessment time points, both randomized groups were evaluated using the same instruments, instructions, equipment, scenarios, and scoring frameworks, as applicable. Cognitive and self-reported measures were administered separately from simulation-based performance assessments. For the LP procedural-performance OSCE, two faculty assessors blinded to randomized group allocation used the same 14-item checklist and scoring framework.

The LP procedural-performance OSCE and simulated vasovagal reaction management assessment were separate outcome measures. The OSCE evaluated predefined LP procedural tasks within the simulation environment, whereas the vasovagal assessment evaluated responses to one standardized simulated intraprocedural event. The vasovagal management score did not contribute to the 0–28 OSCE total.

The simulated vasovagal reaction represented one predefined intraprocedural event and was not intended to indicate its relative frequency, severity, or educational priority among LP-related complications. The assessment did not constitute a comprehensive evaluation of LP complication management; its

findings therefore apply only to performance within the standardized simulated scenario.

The ultrasound sonoanatomy assessment evaluated recognition and interpretation of standardized lumbar ultrasound images relevant to preprocedural localization. It did not assess probe handling, independent image acquisition or optimization, real-time ultrasound guidance of needle advancement, or patient-based ultrasound-assisted LP performance. Within UIS-LP, ultrasound was used for lumbar sonoanatomy training and preprocedural localization rather than real-time needle guidance.

Ultrasound sonoanatomy identification and simulated vasovagal reaction management were assessed only after the educational conditions and were closely aligned with UIS-LP content. Their findings therefore describe performance on the specified post-intervention assessments rather than change from baseline or transfer beyond the trained tasks.

LP procedural performance at 2–4 weeks is reported as procedural performance at reassessment rather than skill retention. The same standardized 14-item OSCE framework was used at the immediate and 2–4-week assessments, and the exact participant-level interval within the prespecified reassessment window was not retained. Testing or recall effects associated with repeated use of the same assessment framework cannot be excluded. Accordingly, the reassessment should not be interpreted independently as evidence of differential skill retention or long-term durability of procedural performance.

UIS-LP and SDL differed in educational format and exposure. UIS-LP was a 2-hour instructor-led multicomponent program incorporating lumbar sonoanatomy training, preprocedural ultrasound localization, supervised LP simulation, repeated practice, feedback, debriefing, and simulated vasovagal reaction management. SDL consisted of 2 weeks of independent access to standardized educational materials and did not include matched instructor contact or hands-on procedural exposure. The comparison therefore concerns the multicomponent UIS-LP program as delivered relative to SDL and does not isolate the contribution of ultrasound or any other individual program component.

Individual SDL study time and electronic resource use were not recorded. Within UIS-LP, individual hands-on training time, number of ultrasound scans, and number of simulated LP attempts were not prospectively documented, and completion was not based on a predefined proficiency threshold. Additional LP- or ultrasound-related educational exposure outside the allocated conditions and cross-group contamination were not systematically assessed.

All objectively assessed procedural outcomes were obtained within a standardized simulation environment. Patient-based LP performance and subsequent clinical LP exposure were not prospectively evaluated. Accordingly, the supplementary assessments should not be interpreted as measures of independent clinical competence, transfer to patient-based LP performance, or improved patient safety.

Organization of the Supplementary Assessment Package

- **Supplementary Table S1. Procedural Knowledge Assessment** — 9-item assessment of LP indications, contraindications, anatomical landmarks, procedural sequence, aseptic technique, and safety; total score, 0–9.
- **Supplementary Table S2. Self-Confidence Questionnaire** — 10-item self-reported questionnaire using a 5-point Likert scale; total score, 10–50.
- **Supplementary Table S3. Ultrasound Sonoanatomy Identification Assessment** — 10-item image-based assessment of lumbar sonoanatomy relevant to preprocedural localization; total score, 0–10.
- **Supplementary Table S4. Standardized Lumbar Puncture OSCE Procedural-Performance Checklist** — 14-item simulation-based procedural-performance assessment using item-specific 0–2 behavioral scoring criteria; total score, 0–28.
- **Supplementary Table S5. Standardized Simulated Vasovagal Reaction Management Checklist and Scenario** — 7-item assessment of responses to a predefined simulated intraprocedural event using item-specific 0–2 behavioral scoring criteria; total score, 0–14.

Supplementary Table S1. Procedural Knowledge Assessment

Purpose

The procedural knowledge assessment evaluated knowledge relevant to lumbar puncture (LP), including lumbar anatomy, procedural preparation and sequencing, aseptic practice, preprocedural ultrasound localization, patient selection and contraindications, procedural safety, and recognition and initial management of a simulated vasovagal reaction.

The assessment comprised nine single-best-answer multiple-choice questions, each with four response options and one predefined correct answer. Items were categorized by cognitive demand as two foundational, three applied, and four

advanced items. This classification described the assessment content and did not affect item weighting or scoring.

Assessment Items and Answer Key

Level	No.	Question	Response Options	Correct Answer
Foundational	1	A patient is positioned for LP. A line joining the highest points of the iliac crests is used to assist landmark identification. Which vertebral landmark does this line most commonly approximate?	A. L1 spinous process; B. L2–L3 interspace; C. L4 spinous process/L4–L5 region; D. S1 spinous process	C
Foundational	2	Why is LP generally performed below the level at which the adult spinal cord terminates?	A. To avoid the epidural venous plexus completely; B. To reduce the risk of direct spinal cord injury; C. To prevent post-dural puncture headache; D. To ensure that cerebrospinal fluid pressure cannot increase during the procedure	B
Applied	3	During preprocedural ultrasound scanning, the learner identifies adjacent hyperechoic bony structures with posterior acoustic shadowing and an intervening acoustic window. Which finding is most useful for planning	A. The center of the bony acoustic shadow; B. The interspinous/interlaminar window between adjacent bony landmarks; C. The most superficial hyperechoic structure regardless of level; D. The lateral border of the paraspinal muscle	B

Level	No.	Question	Response Options	Correct Answer
		the needle-entry site?		
Applied	4	After skin preparation and application of sterile gloves, the operator touches a nonsterile ultrasound control panel immediately before needle insertion. What is the most appropriate next action?	A. Continue because the puncture site remains sterile; B. Wipe the gloves with antiseptic and proceed; C. Re-establish sterility before continuing the procedure; D. Continue if a sterile needle has not yet been touched	C
Applied	5	During an attempted LP, the needle appears to have advanced an appropriate distance, but no cerebrospinal fluid is obtained. The patient remains stable and no neurological symptoms occur. What is the most appropriate initial response?	A. Advance the needle substantially farther without reassessment; B. Reassess positioning, anatomical landmarks, and needle trajectory before another attempt; C. Rotate the needle repeatedly while maintaining the same trajectory; D. Abandon the procedure immediately without reassessing technique	B
Advanced	6	A patient with suspected meningitis has papilledema and a reduced level of consciousness. Which approach is most appropriate	A. Perform LP immediately because cerebrospinal fluid analysis has diagnostic priority; B. Assess for raised intracranial pressure and the need for neuroimaging before LP; C. Perform LP only in the sitting position; D. Use	B

Level	No.	Question	Response Options	Correct Answer
		before proceeding with LP?	ultrasound localization because it eliminates the neurological concern	
Advanced	7	During LP, a patient becomes pale and diaphoretic and reports nausea and light-headedness. Heart rate decreases from 82 to 48 beats/min and blood pressure falls. What is the most likely explanation?	A. Local anesthetic systemic toxicity; B. Vasovagal reaction; C. Post-dural puncture headache; D. Traumatic LP	B
Advanced	8	During the same episode, the patient becomes increasingly hypotensive and briefly loses consciousness. What should be the immediate priority?	A. Complete cerebrospinal fluid collection before managing the event; B. Sit the patient upright and continue observation; C. Stop the procedure, position the patient safely, assess airway and circulation, and initiate appropriate supportive management; D. Remove the needle and immediately repeat the procedure at another level	C
Advanced	9	A learner correctly identifies the intended puncture level using ultrasound and completes the needle-insertion steps but does not recognize progressive	A. Ultrasound anatomical identification; B. Needle-insertion technique; C. Patient monitoring and recognition of clinical deterioration; D. Knowledge of lumbar surface anatomy	C

Level	No.	Question	Response Options	Correct Answer
		diaphoresis, bradycardia, and hypotension in the simulated patient. Which aspect of procedural performance most clearly requires attention?		

Administration

The procedural knowledge assessment was administered at two predefined time points: baseline, before randomization, and immediately after completion of the allocated educational condition. The same nine items, response options, and scoring criteria were used at both assessments. Participants were not provided with the answer key or item-level corrective feedback between the baseline and immediate post-intervention assessments.

Scoring

Each correct response received 1 point and each incorrect response received 0 points, producing a total score ranging from 0 to 9. All nine items contributed equally to the total score, irrespective of cognitive-level classification. Higher scores indicated a greater number of correct responses.

Percentage-of-maximum values were used only for descriptive graphical presentation. Statistical analyses were conducted using the original 0–9 scores.

Interpretation

The total score represented performance on a cognitive assessment aligned with the predefined LP learning objectives and was analyzed as a procedural knowledge outcome. It was distinct from directly observed LP procedural performance assessed using the 14-item OSCE. Baseline procedural knowledge therefore did not constitute a baseline measure or proxy for OSCE procedural performance. Because the OSCE was not administered at baseline, directly observed baseline procedural performance was unavailable.

The ultrasound-related content addressed preprocedural anatomical localization and planning. The assessment did not evaluate practical probe handling, independent image acquisition or optimization, real-time ultrasound

guidance of needle advancement, or patient-based ultrasound-assisted LP performance.

The vasovagal-related items assessed recognition and initial management of the predefined simulated event and did not represent comprehensive assessment of LP-related complications or broader clinical management.

Instrument Development and Content Review

The procedural knowledge assessment underwent expert content review and pilot testing before participant enrollment as part of the study instrument-development process. Review addressed item relevance, clarity, suitability for clinical-year medical students, and alignment with the predefined learning objectives.

No instrument-specific quantitative validity or reliability estimate is reported because a verified estimate was unavailable in the retained study records. No quantitative validity or reliability values were retrospectively calculated or assigned.

Supplementary Table S2. Self-Confidence Questionnaire

Purpose

The self-confidence questionnaire assessed participants' perceived confidence in activities relevant to lumbar puncture (LP). The instrument comprised 10 items organized across five domains: anatomical landmark identification, patient positioning, procedural steps, contraindications and procedural safety, and aseptic technique.

The questionnaire was a self-reported educational measure and was distinct from objectively assessed LP procedural performance using the Objective Structured Clinical Examination (OSCE).

Assessment Items

Domain	Item	Statement
Anatomical landmark identification	1	I feel confident identifying lumbar anatomical landmarks relevant to lumbar puncture.
	2	I feel confident using ultrasound-based anatomical landmark identification to determine an appropriate lumbar puncture site.

Domain	Item	Statement
Patient positioning	3	I feel confident positioning a patient appropriately for lumbar puncture.
	4	I feel confident recognizing and correcting patient-positioning factors that may make lumbar puncture more difficult.
Procedural steps	5	I feel confident preparing the equipment and materials required for lumbar puncture.
	6	I feel confident performing the steps of lumbar puncture in the correct sequence.
Contraindications and procedural safety	7	I feel confident identifying important contraindications to lumbar puncture before beginning the procedure.
	8	I feel confident recognizing procedure-related complications, including a vasovagal reaction, and initiating appropriate immediate management.
Aseptic technique	9	I feel confident establishing appropriate sterile conditions before lumbar puncture.
	10	I feel confident maintaining aseptic technique throughout the lumbar puncture procedure.

Response Scale

Each item was rated using a 5-point Likert scale:

Score	Response
1	Strongly disagree
2	Disagree
3	Neutral
4	Agree
5	Strongly agree

Administration

The self-confidence questionnaire was administered at two predefined time points: baseline, before randomization, and immediately after completion of the allocated educational condition. The same 10 items, response options, instructions, and scoring procedure were used at both assessments.

The questionnaire was administered separately from the simulation-based performance assessments. Self-confidence scores did not contribute to either the 14-item LP procedural-performance OSCE score or the simulated vasovagal reaction management score. Self-confidence was not reassessed at 2–4 weeks.

Scoring

Each item was scored from 1 to 5 according to the selected response. The 10 item scores were summed to produce a total score ranging from 10 to 50, with higher scores indicating greater self-reported confidence in the assessed LP-related activities. All items contributed equally to the total score.

The five domains were:

- **Anatomical landmark identification:** Items 1–2
- **Patient positioning:** Items 3–4
- **Procedural steps:** Items 5–6
- **Contraindications and procedural safety:** Items 7–8
- **Aseptic technique:** Items 9–10

The prespecified self-confidence outcome was the 10-item total score. Domain-level values were descriptive and were not analyzed as separate inferential outcomes.

Interpretation

The total score represented participants' self-reported confidence in the LP-related activities included in the questionnaire and was analyzed separately from directly observed LP procedural performance. Higher self-confidence scores should not be interpreted as evidence of objectively demonstrated procedural performance, independent clinical competence, or patient-based LP performance.

The ultrasound-related item addressed confidence in anatomical landmark identification for selection of an intended LP site. It did not assess confidence in practical image acquisition, probe handling, image optimization, or real-time ultrasound guidance of needle advancement.

The procedural-safety domain included recognition and initial management of a vasovagal reaction as one component of the questionnaire and did not constitute a comprehensive assessment of confidence in managing LP-related complications.

Content Validity and Internal Consistency

The self-confidence questionnaire underwent expert content review and pilot testing before participant enrollment as part of the study instrument-development process. Expert review addressed item relevance, clarity, suitability for clinical-year medical students, and alignment with the intended assessment domains.

The documented scale-level Content Validity Index using the average method (S-CVI/Ave) was **0.96**, and internal consistency was **Cronbach's α = 0.81**. Complete item-level Content Validity Index (I-CVI) records were unavailable for verification during manuscript revision; therefore, unsupported item-level validity estimates were not retrospectively reconstructed or calculated.

Supplementary Table S3. Ultrasound Sonoanatomy Identification Assessment

Purpose

The ultrasound sonoanatomy identification assessment evaluated participants' ability to identify and interpret lumbar ultrasound anatomy relevant to preprocedural lumbar puncture (LP) localization and planning. The assessment focused on recognition of selected osseous and neuraxial structures, relevant acoustic windows, and sonographic features used for anatomical localization.

The assessment comprised 10 structured image-based items using four standardized lumbar ultrasound figures. Items were categorized by intended complexity as three basic, four intermediate, and three advanced items. This classification described the assessment content and did not affect item weighting or scoring.

The assessment evaluated image-based ultrasound sonoanatomy identification and interpretation. It did not assess probe handling, independent image acquisition or optimization, real-time ultrasound guidance of needle advancement, or patient-based ultrasound-assisted LP performance.

Image Source

The lumbar ultrasound figures used in the assessment were obtained from the NYSORA educational resource *Spinal Sonography and Applications of*

Ultrasound for Central Neuraxial Blocks, authored by Manoj K. Karmakar and Ki Jinn Chin.

The figures are included to document the image-based assessment used in the study. Appropriate source attribution should accompany reproduced figures, and permission for reproduction or modification should be obtained from the copyright holder where required. For presentation in this supplementary material, the source images are designated Figures 1–4.

Ultrasound Figures Used in the Assessment

Figure	Ultrasound View	Principal Anatomical and Sonographic Features Represented
**Figure 1**	Paramedian sagittal oblique lumbar view	Laminae, interlaminar spaces, ligamentum flavum, posterior dura, epidural space, intrathecal space, and anterior complex
**Figure 2**	Median sagittal lumbar view	Spinous processes, interspinous spaces, and associated acoustic shadowing
**Figure 3**	Paramedian sagittal transverse-process view	Transverse processes and their acoustic shadows, psoas muscle, and characteristic trident sign
**Figure 4**	Transverse spinous-process view	Spinous process, laminae, and posterior acoustic shadowing from bony structures limiting visualization of the spinal canal and deeper neuraxial structures

Assessment Structure

Complexity	Item	Figure	Assessment Focus
Basic	1	Figure 2	Spinous process
Basic	2	Figure 2	Interspinous space
Basic	3	Figure 1	Lamina
Intermediate	4	Figure 1	Interlaminar space
Intermediate	5	Figure 3	Transverse process
Intermediate	6	Figure 3	Psoas muscle

Complexity	Item	Figure	Assessment Focus
Intermediate	7	Figure 4	Posterior acoustic shadowing limiting visualization of deeper neuraxial structures
Advanced	8	Figure 1	Intrathecal space
Advanced	9	Figure 1	Interlaminar acoustic window relevant to LP planning
Advanced	10	Figures 1 and 4	Comparison of sonographic views to identify the view providing an interlaminar acoustic window for visualization of deeper neuraxial structures relevant to LP planning

Image Presentation and Response Format

For items requiring identification of a specific anatomical structure or space, a white arrow indicated the target to be identified. When the same figure was used for multiple items, a question-specific version was presented with the arrow identifying only the structure or space relevant to that item. Interpretation-based items used the overall sonographic appearance or comparison between figures, as specified by the assessment task.

Responses were recorded using either direct anatomical identification or predefined response options, depending on the item. Both randomized groups received the same figures, questions, response formats, and scoring criteria. Anatomical labels, explanatory descriptions, and predefined correct responses presented in this supplementary material constitute the answer key and were not displayed during the scored assessment.

Administration

The ultrasound sonoanatomy identification assessment was administered once, immediately after completion of the allocated educational condition. No baseline ultrasound sonoanatomy assessment was performed.

Both randomized groups completed the same 10-item assessment under standardized conditions. The planned completion time was approximately 5 minutes. The assessment was administered separately from the 14-item simulation-based LP procedural-performance OSCE, and no corrective feedback or answer key was provided during the scored assessment.

Scoring

Each item had one predefined correct response. Correct responses received 1 point and incorrect responses received 0 points, producing a total score ranging from 0 to 10. Higher scores indicated a greater number of correct responses on the standardized image-based assessment.

All 10 items contributed equally to the total score. Classification as basic, intermediate, or advanced described the intended complexity of the assessment tasks and did not affect item weighting. The distribution was three basic items (Items 1–3), four intermediate items (Items 4–7), and three advanced items (Items 8–10). Separate scores for these categories were not analyzed as study outcomes; the total 0–10 score constituted the ultrasound sonoanatomy identification outcome.

Interpretation

The total score represented performance on a standardized image-based assessment of lumbar ultrasound sonoanatomy identification and interpretation relevant to preprocedural LP localization. It did not assess practical ultrasound skills, independent image acquisition or optimization, real-time ultrasound guidance of needle advancement, or patient-based ultrasound-assisted LP performance.

Because ultrasound sonoanatomy identification was not assessed at baseline, the immediate post-intervention result represents a post-intervention between-group comparison rather than change from baseline. The outcome should therefore be interpreted as performance on the specified image-based assessment rather than evidence of broader ultrasound competence or independent clinical performance.

Relationship to the UIS-LP Educational Condition

Lumbar sonoanatomy identification and preprocedural ultrasound localization were components of the multicomponent UIS-LP program. The ultrasound sonoanatomy assessment was therefore closely aligned with content included in the UIS-LP educational condition.

The observed between-group difference should be interpreted within this context and does not isolate the contribution of ultrasound from supervised simulation, repeated practice, feedback, debriefing, instructor contact, or other components of UIS-LP. Performance on the standardized images also does not establish transfer to unfamiliar ultrasound images, different anatomical presentations, practical image acquisition, real-time needle guidance, or patient-based LP performance.

Content Review and Pilot Testing

The ultrasound sonoanatomy identification assessment underwent expert content review and pilot testing before participant enrollment as part of the study instrument-development process. Review addressed item relevance, clarity, suitability for clinical-year medical students, alignment with the predefined learning objectives, and suitability of the selected ultrasound images for the assessment tasks.

Pilot testing evaluated clarity, feasibility, administration time, image presentation, and application of the scoring framework. Clarifications identified during this process were incorporated before participant enrollment and formal data collection.

Complete item-level quantitative content-validity records and a separate internal-consistency estimate were unavailable in the retained study records. Accordingly, unsupported Content Validity Index or reliability estimates were not retrospectively reconstructed or calculated.

Statistical and Reporting Considerations

Ultrasound sonoanatomy identification was an exploratory secondary outcome, distinct from the prespecified primary LP procedural-performance OSCE. A post hoc Bonferroni sensitivity analysis was applied across the four immediate secondary outcomes using a threshold of $\alpha = 0.0125$ (0.05/4). The ultrasound sonoanatomy comparison remained below this threshold; however, its exploratory status was unchanged.

Interpretation should account for the absence of a baseline ultrasound sonoanatomy assessment and the close alignment between the assessment and UIS-LP content. The finding therefore represents performance on the specified image-based assessment and should not be interpreted as evidence of broader ultrasound competence, independent clinical readiness, or patient-based LP performance.

Abbreviations: LP, lumbar puncture; OSCE, Objective Structured Clinical Examination; UIS-LP, ultrasound-integrated simulation-based lumbar puncture.

Figure S1 — Paramedian sagittal oblique view

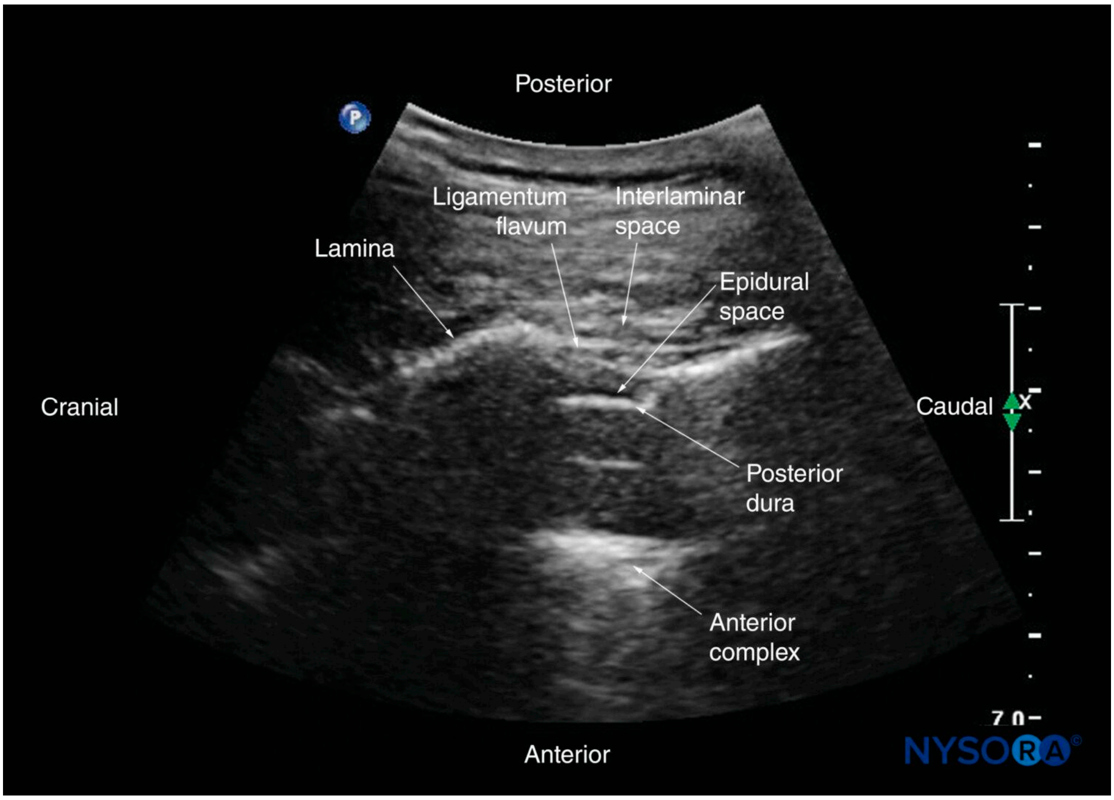
Posterior
Ligamentum flavum
Interlaminar space
Lamina
Epidural space
Cranial
Caudal
Posterior dura
Anterior complex
7.0
Anterior
NYSORA

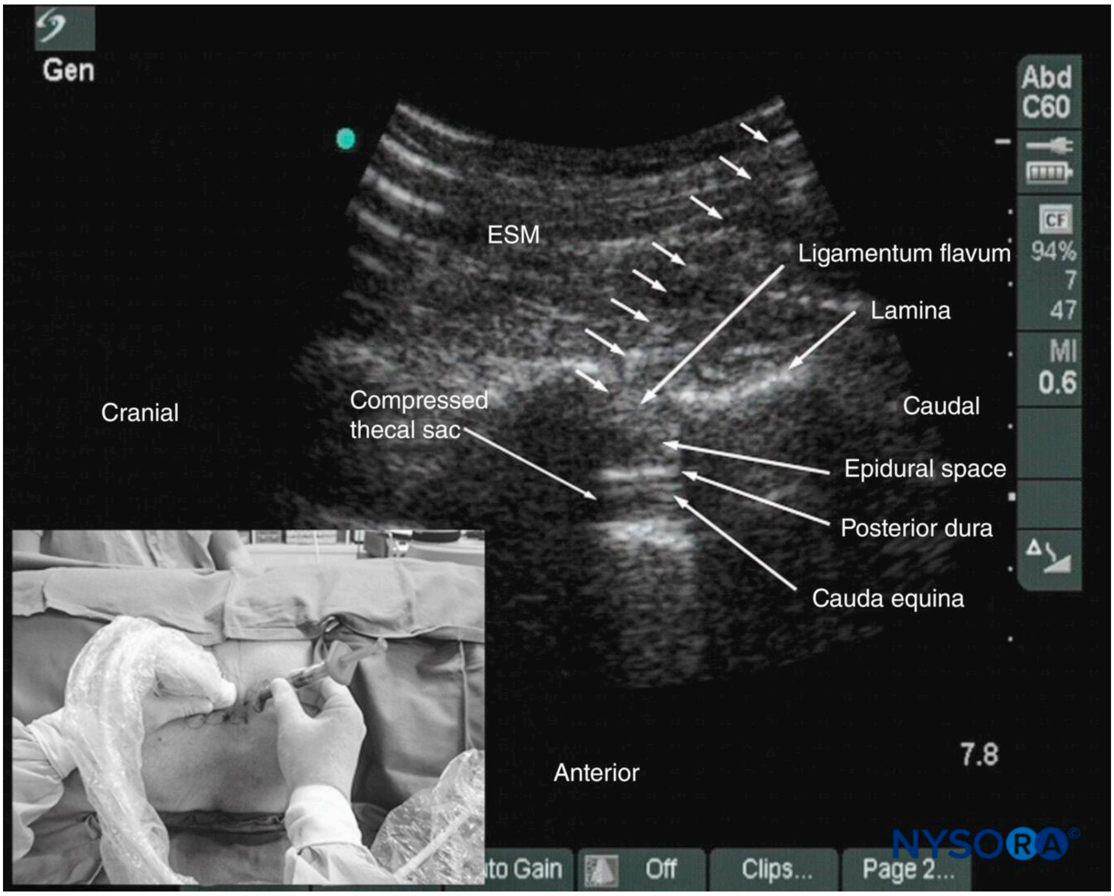
Gen
Abd C60
ESM
Ligamentum flavum
94%
7
47
Lamina
MI 0.6
Cranial
Compressed thecal sac
Caudal
Epidural space
Posterior dura
Cauda equina
7.8
Anterior
NYSORA
Off
Clips...
Page 2...

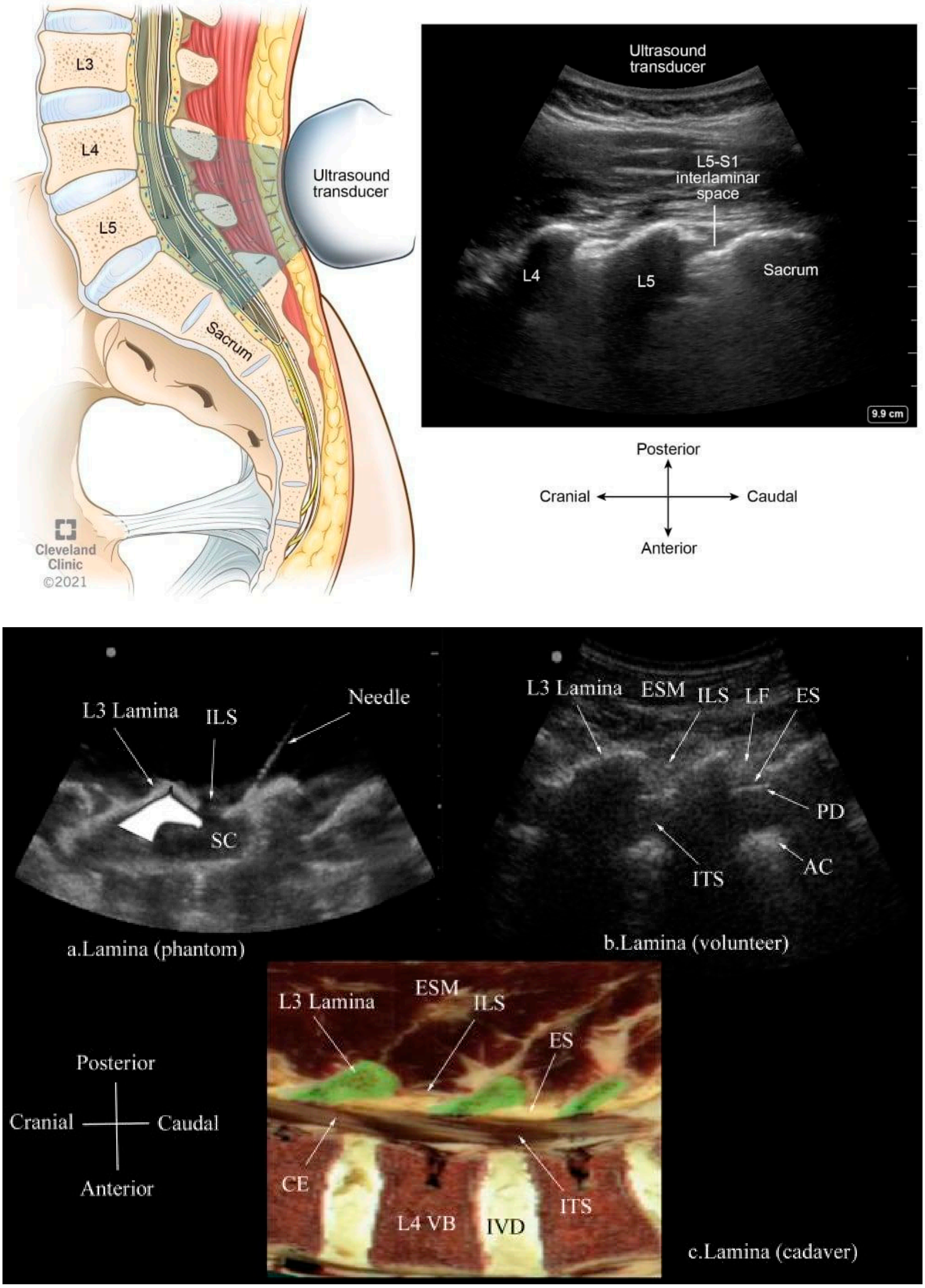

Used for Items 3, 4, 8, and 9, and for the comparison in **Item 10**.

Figure S2 — Median sagittal view

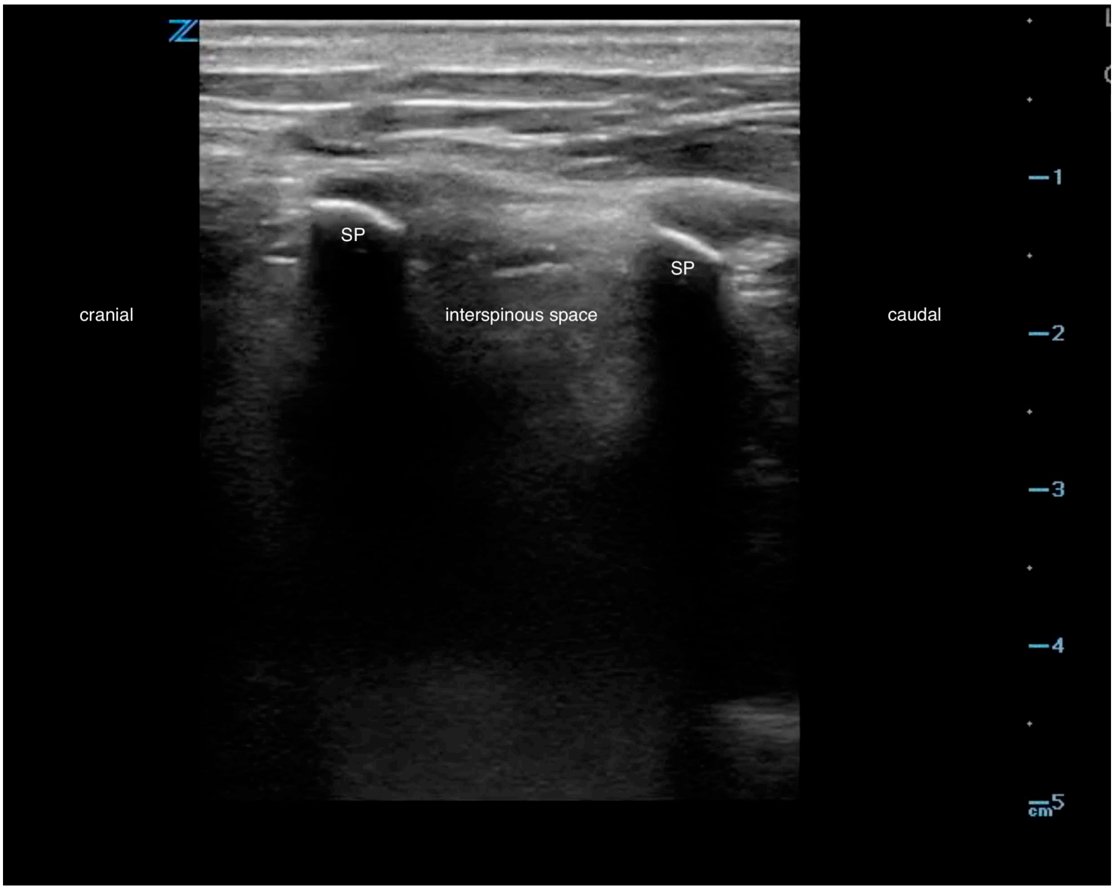
SP
SP
cranial
interspinous space
caudal
1
2
3
4
cm 5

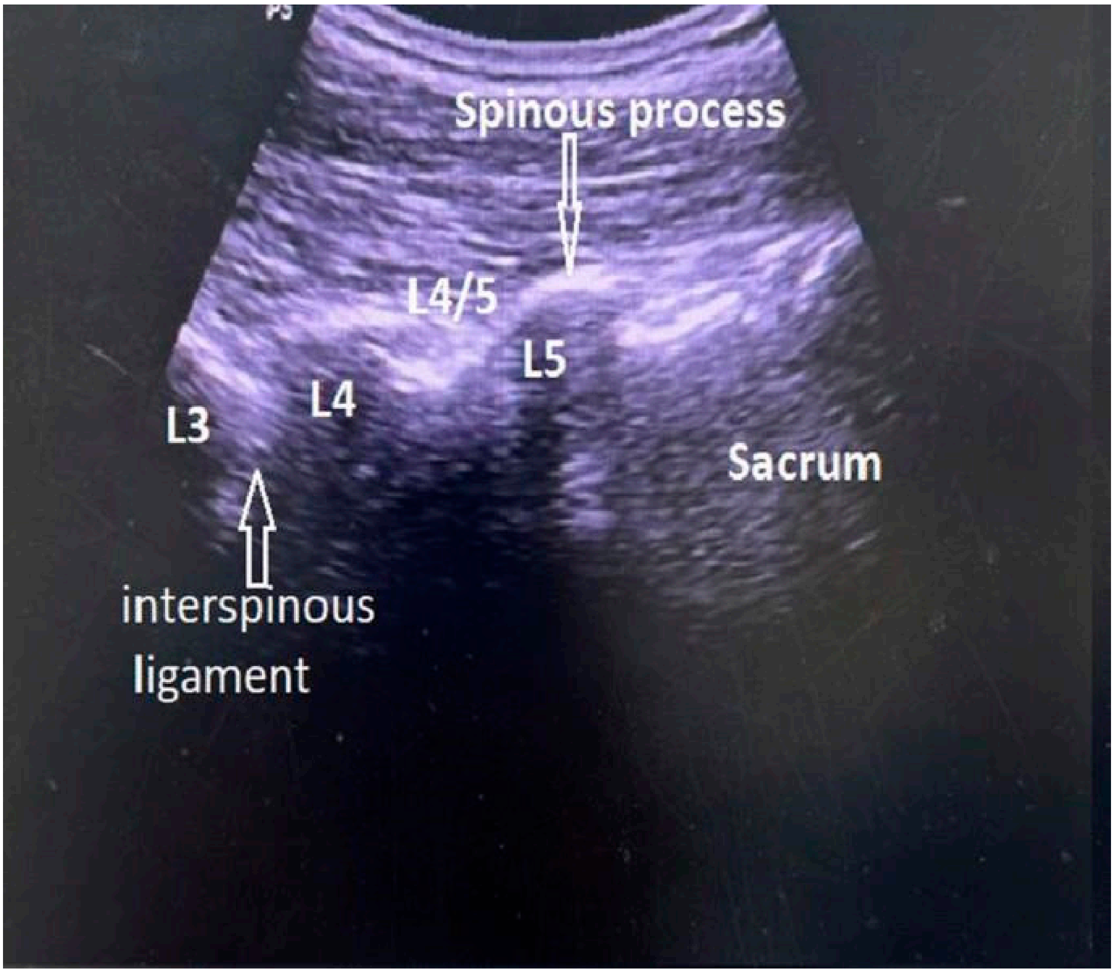
Spinous process
L4/5
L5
L4
L3
Sacrum
interspinous
ligament

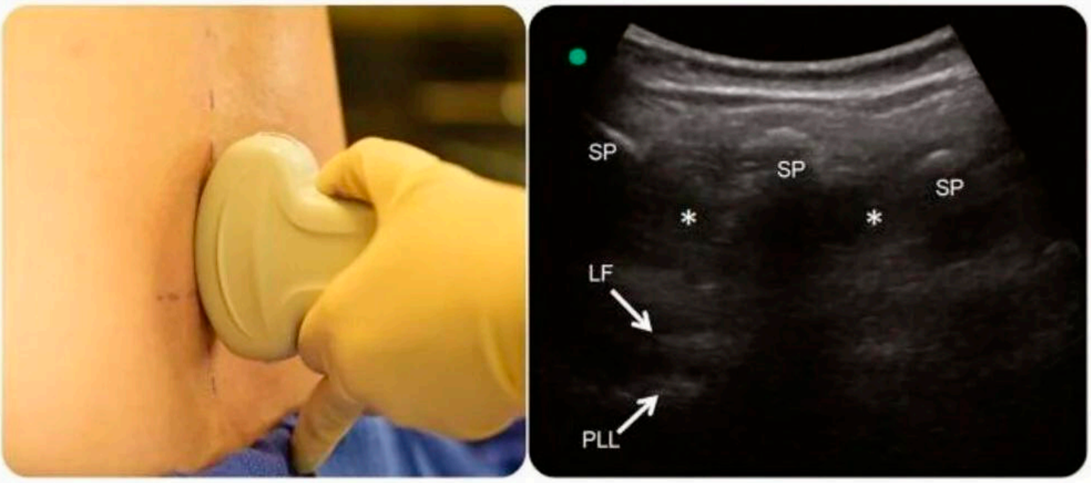

SP: spinous process, LF: ligamentum flavum, PLL: posterior longitudinal ligament, *interspinous spaces[1]

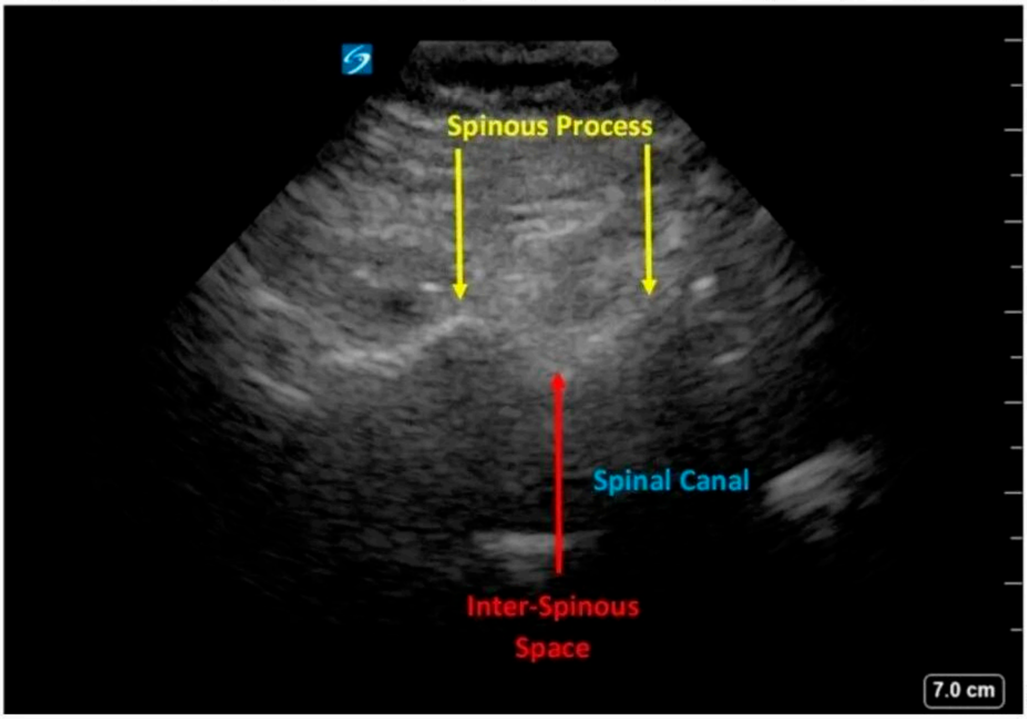

[3]

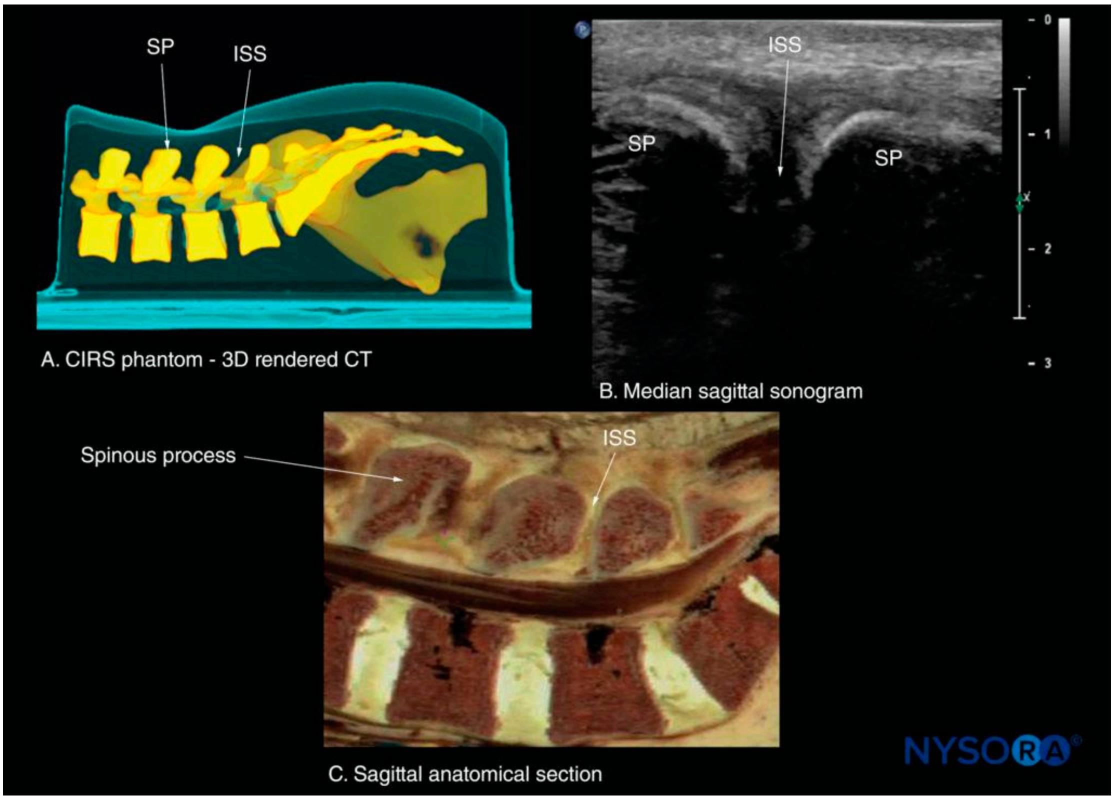

Used for Items 1 and 2.

Figure S3 — Paramedian sagittal transverse-process view

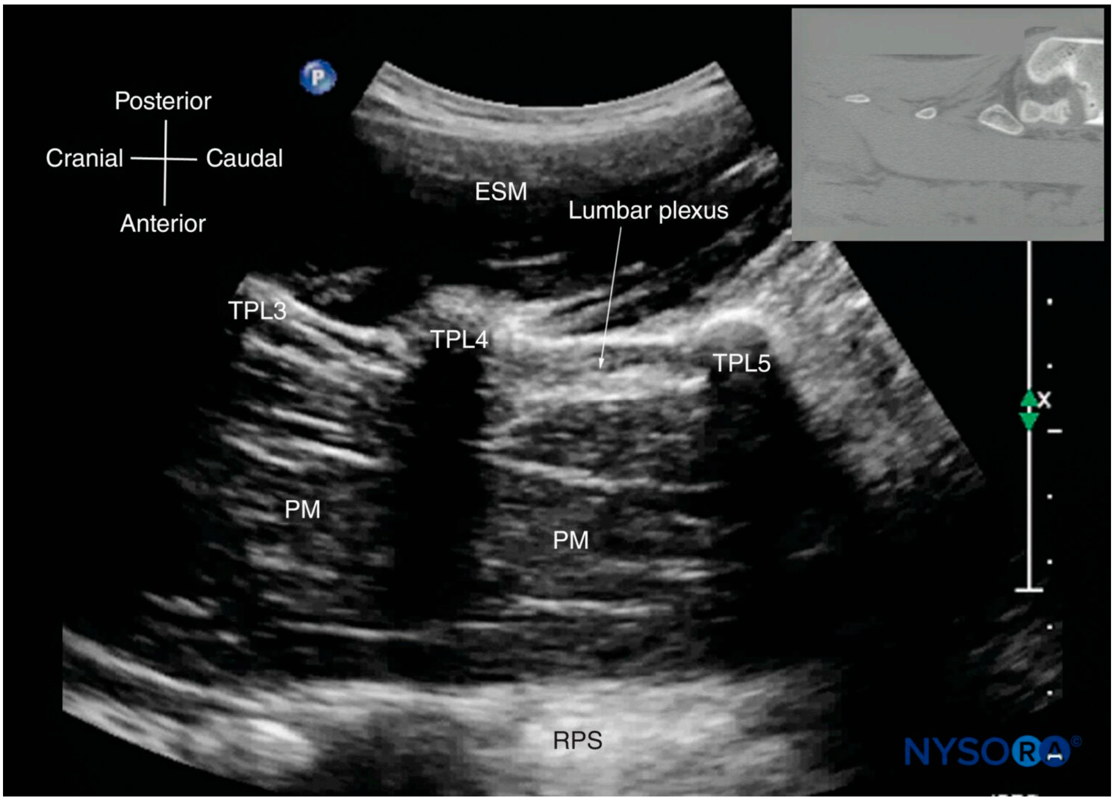

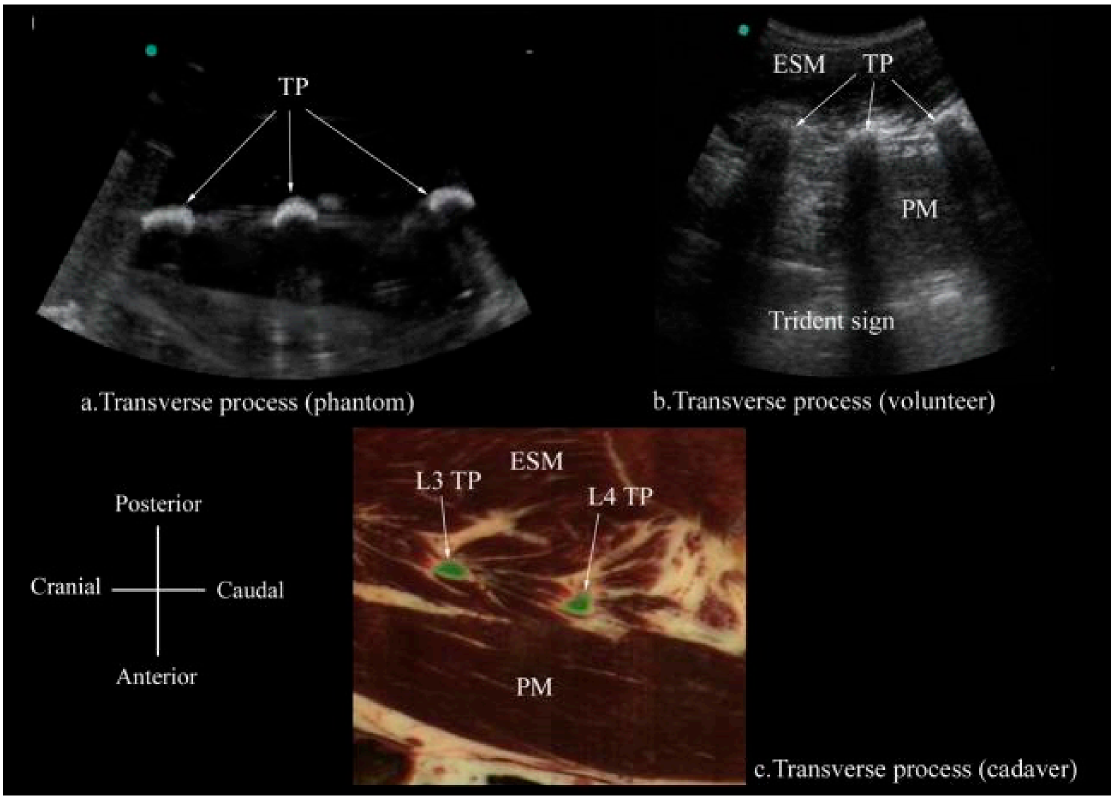
TP
ESM
TP
PM
Trident sign
a.Transverse process (phantom)
b.Transverse process (volunteer)
Posterior
Cranial
Caudal
Anterior
L3 TP
ESM
L4 TP
PM
c.Transverse process (cadaver)

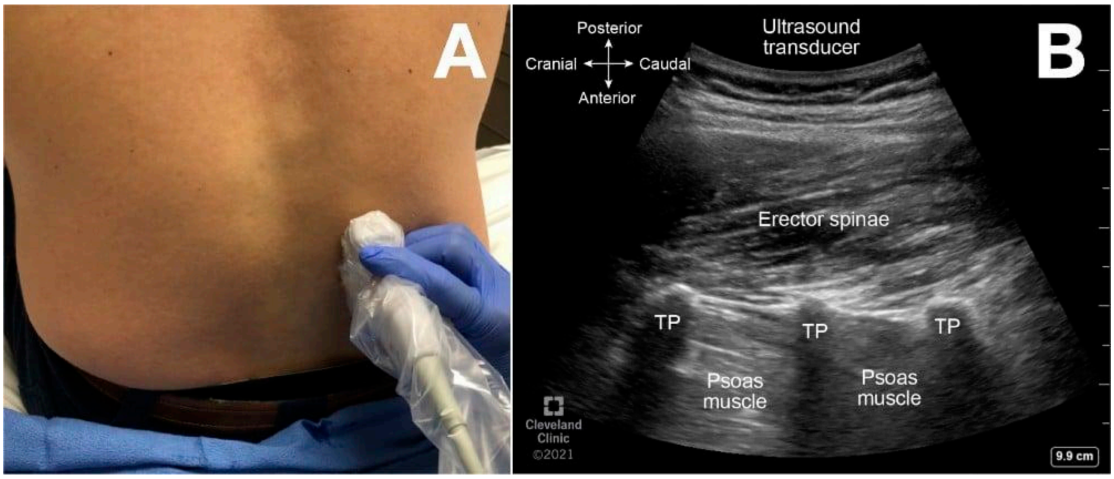
A
B
Posterior
Cranial
Caudal
Anterior
Ultrasound transducer
Erector spinae
TP
TP
TP
Psoas muscle
Psoas muscle
Cleveland Clinic ©2021
9.9 cm

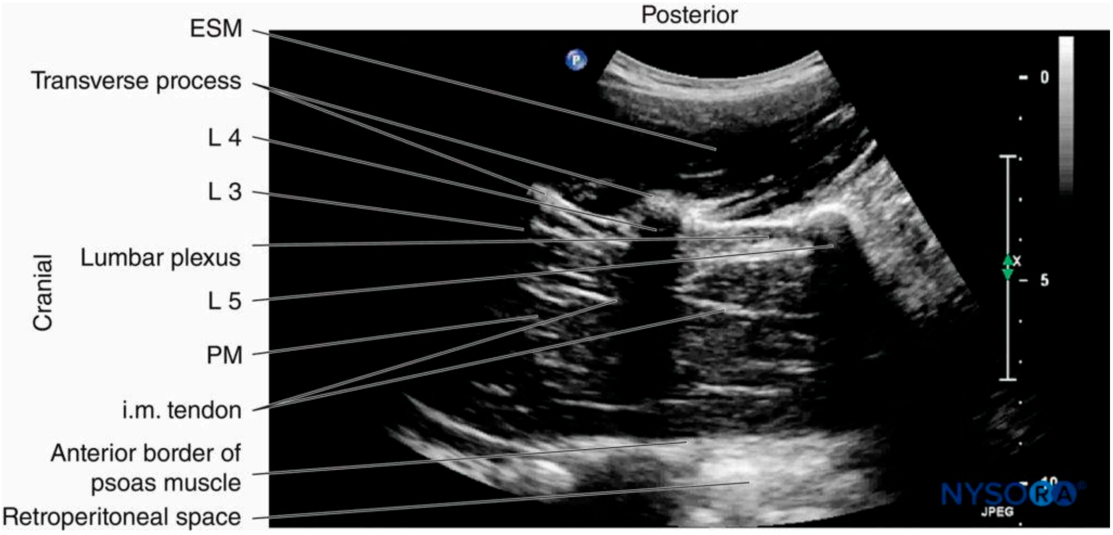
Posterior
ESM
Transverse process
L 4
L 3
Lumbar plexus
L 5
PM
i.m. tendon
Anterior border of psoas muscle
Retroperitoneal space
Cranial
0
5
NYSORA

Used for Items 5 and 6.

Figure S4 — Transverse spinous-process view

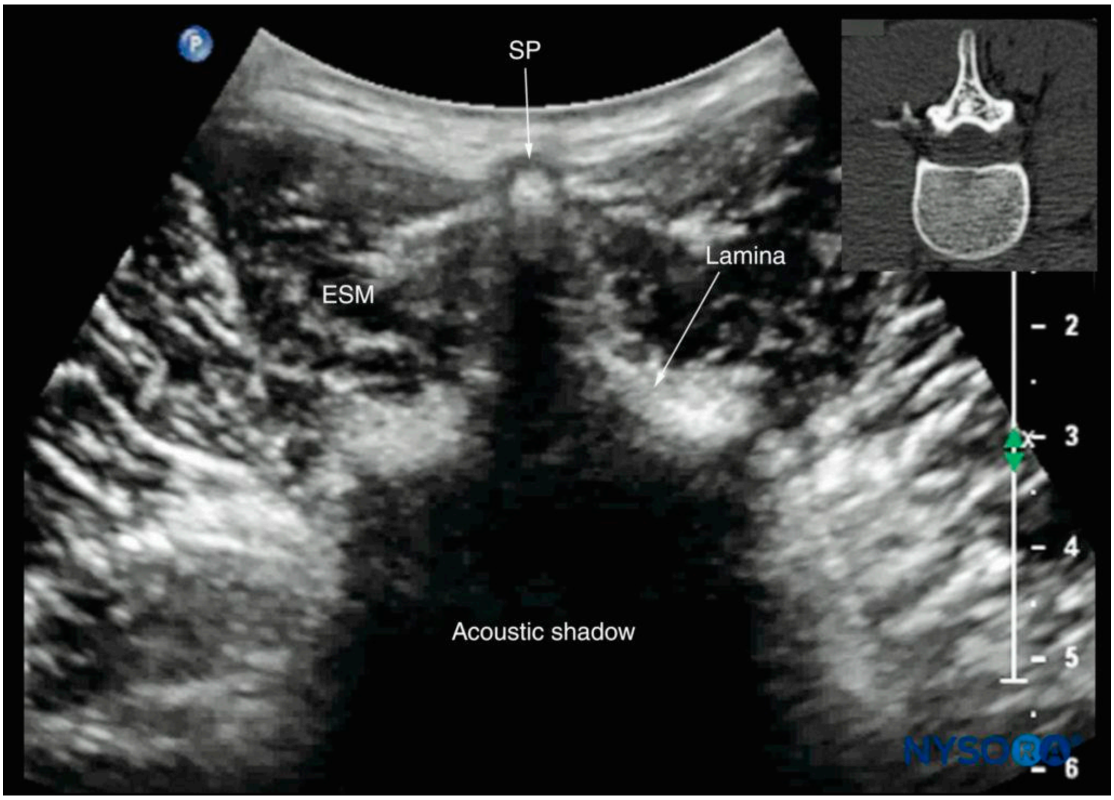

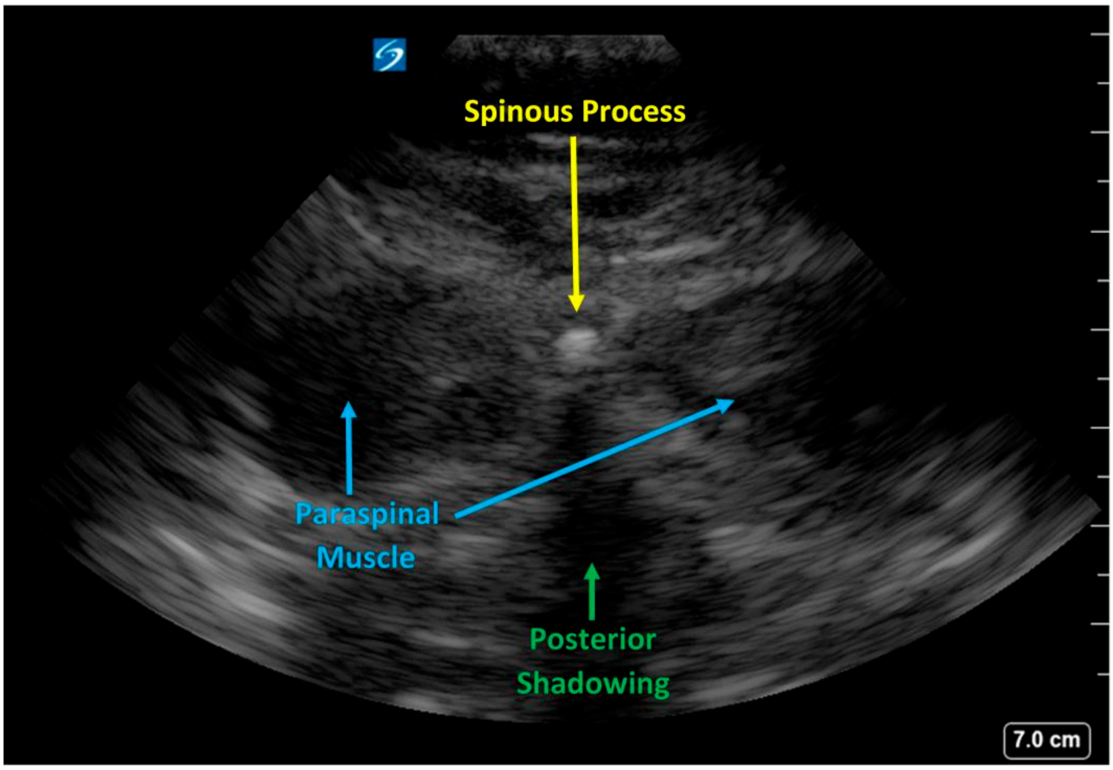

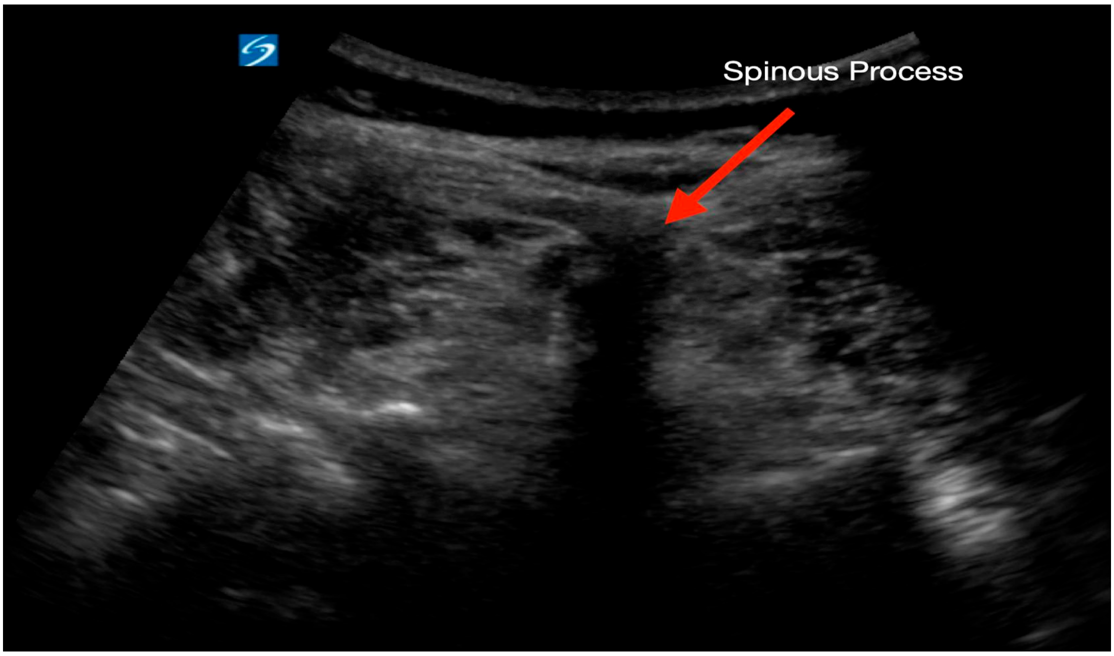
Spinous Process

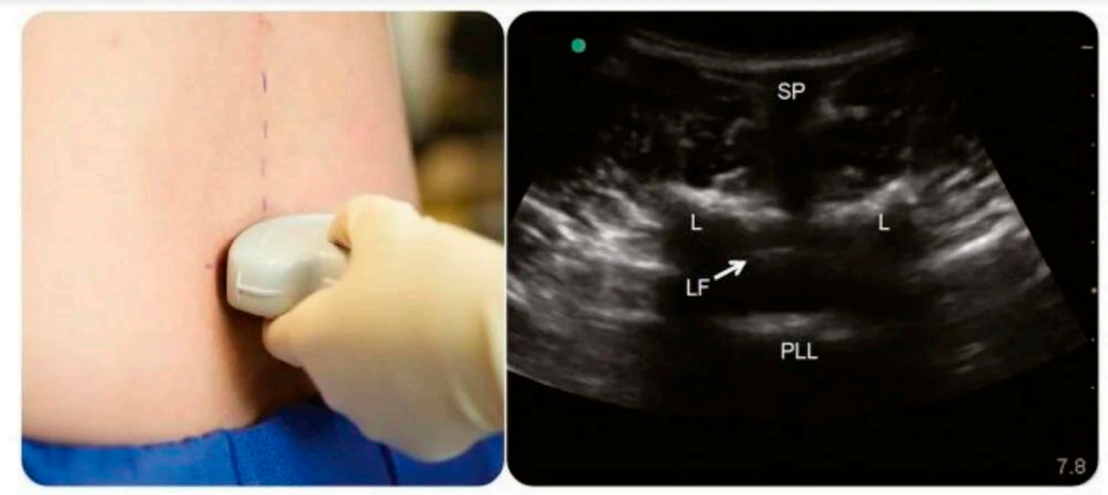

SP: spinal process, L: lamina, LF: ligamentum flavum, PLL: posterior longitudinal ligament[1]

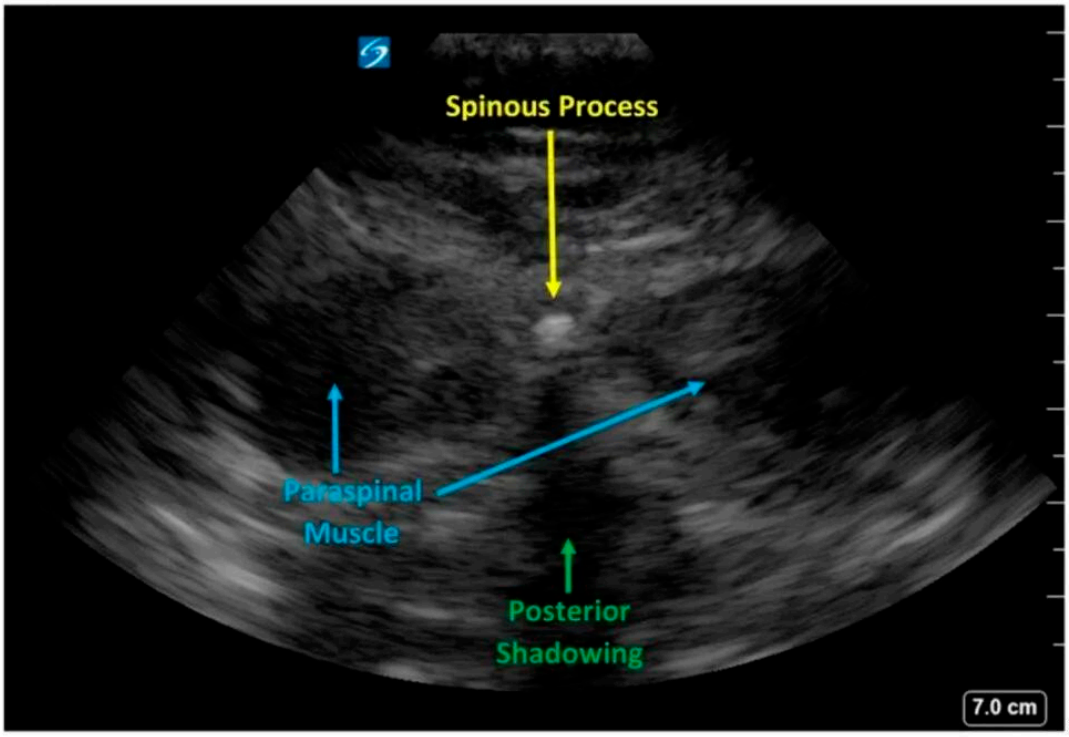

[3]

Used for Item 7 and, together with Figure 1, for **Item 10**.

Supplementary Table S4. Standardized Lumbar Puncture OSCE Procedural-Performance Checklist

Purpose

The Objective Structured Clinical Examination (OSCE) assessed simulation-based lumbar puncture (LP) procedural performance under standardized conditions. The assessment covered patient preparation and communication, positioning, anatomical landmark identification, preprocedural ultrasound localization, equipment preparation, aseptic technique, needle insertion and

handling, procedural sequencing, response to procedural concerns, and immediate post-procedural care.

The OSCE comprised 14 equally weighted items, each scored from 0 to 2 points, yielding a total score ranging from 0 to 28. The total score represented performance on the predefined simulation-based LP tasks.

The OSCE was distinct from the separate simulated vasovagal reaction management assessment.

Standardized Assessment Scenario

Participants were instructed to perform a diagnostic LP on a clinically stable simulated adult patient undergoing evaluation for suspected meningitis. For the purposes of the station, the simulated patient had undergone appropriate clinical assessment, no contraindication requiring postponement or additional investigation was present, and LP had been determined appropriate to proceed.

The station focused on performance of the standardized LP procedure rather than determination of whether LP was clinically indicated. Participants were expected to demonstrate appropriate communication, positioning, anatomical localization, preprocedural ultrasound localization, equipment preparation, aseptic technique, needle insertion and handling, procedural sequencing, response to procedural concerns, and immediate post-procedural care.

Ultrasound was used for preprocedural anatomical localization and selection of the intended puncture site. Real-time ultrasound visualization or guidance of needle advancement was not assessed.

Standardized Candidate Instruction

You are asked to perform a lumbar puncture on a clinically stable adult patient undergoing diagnostic evaluation for suspected meningitis. The patient has been clinically assessed, has no identified contraindication to lumbar puncture, and the procedure has been authorized to proceed. Please perform the procedure safely and systematically while maintaining appropriate patient communication and procedural safety.

The same scenario, simulation environment, equipment, candidate instructions, checklist, and scoring framework were used for both randomized groups.

Item-Specific Behavioral Anchors

Item	Assessed Behavior	0 Points	1 Point	2 Points
**1**	Hand hygiene	Does not perform hand hygiene.	Performs hand hygiene only after prompting or at an inappropriate stage.	Independently performs hand hygiene appropriately before procedural preparation.
**2**	Patient identification, introduction, and explanation	Does not appropriately introduce self, confirm patient identity, or explain the procedure.	Completes some but not all required elements or requires prompting.	Independently introduces self, confirms patient identity, and appropriately explains the procedure.
**3**	Patient positioning	Positions the patient inappropriately for LP.	Selects an appropriate position but requires prompting or correction to optimize positioning.	Independently positions the patient appropriately for LP and optimizes lumbar access.
**4**	Anatomical landmark identification	Does not correctly identify the relevant lumbar landmarks.	Identifies some relevant landmarks but demonstrates uncertainty or requires prompting.	Independently and correctly identifies the relevant lumbar anatomical landmarks and intended interspace.
**5**	Preprocedural ultrasound localization	Does not obtain or appropriately interpret the required lumbar ultrasound view.	Obtains a partially adequate view or requires prompting to identify relevant lumbar anatomy.	Independently obtains an appropriate ultrasound view and correctly identifies the

Item	Assessed Behavior	0 Points	1 Point	2 Points
				relevant lumbar sonoanatomy.
**6**	Identification of intended puncture site	Selects or marks an inappropriate puncture site.	Identifies an acceptable site but requires prompting or correction.	Independently identifies and marks an appropriate intended puncture site based on the anatomical and ultrasound findings.
**7**	Equipment preparation	Omits essential equipment or is unable to prepare the required procedural equipment appropriately.	Prepares most required equipment but has a minor omission or requires prompting.	Independently prepares and organizes the required procedural equipment appropriately.
**8**	Aseptic technique	Fails to establish appropriate asepsis or commits a major breach of aseptic technique.	Generally follows aseptic technique but has a minor correctable omission or requires prompting.	Independently establishes and maintains appropriate aseptic technique throughout the procedure.
**9**	Needle insertion technique and trajectory	Demonstrates an inappropriate or unsafe insertion technique or trajectory.	Demonstrates generally appropriate insertion but requires prompting, assistance, or correction.	Independently performs controlled needle insertion using an appropriate direction and trajectory.

Item	Assessed Behavior	0 Points	1 Point	2 Points
**10**	Procedural sequencing	Performs major procedural steps in an inappropriate sequence.	Completes most procedural steps appropriately but has minor sequencing errors or requires prompting.	Independently performs the procedural steps in an appropriate and logical sequence.
**11**	Needle handling and sharps safety	Demonstrates unsafe needle or sharps handling.	Generally demonstrates safe handling but requires correction for a minor safety issue.	Consistently demonstrates appropriate needle handling and sharps safety.
**12**	Response to patient discomfort or procedural concern	Does not recognize or appropriately respond to patient discomfort or a procedural concern.	Recognizes the concern, but the response is delayed, incomplete, or requires prompting.	Promptly recognizes the concern, pauses or reassesses when appropriate, and responds appropriately within the simulated setting.
**13**	Communication and reassurance	Provides inadequate communication or reassurance during the procedure.	Provides intermittent or incomplete communication or requires prompting.	Communicates clearly and provides appropriate explanation and reassurance throughout the procedure.
**14**	Procedure completion and immediate post-procedural care	Omits essential procedure-completion or	Performs most required steps but incompletely or with prompting.	Independently completes the procedure appropriately and

Item	Assessed Behavior	0 Points	1 Point	2 Points
		immediate post-procedural steps.		provides the required immediate post-procedural care and instructions.

The item-specific behavioral anchors operationalize the original 14-item, 0–2 scoring framework and do not alter the number of items, item weighting, total score range, outcome definition, or participant-level scores used in the analyses.

Scoring

Each item was scored from 0 to 2 points:

- **0 points:** the assessed behavior was absent, incorrect, or inappropriate.
- **1 point:** the behavior was partially or incompletely performed or required prompting or correction.
- **2 points:** the behavior was performed correctly and independently within the standardized simulation setting.

All 14 items were equally weighted and summed to generate a total OSCE score ranging from 0 to 28. The prespecified primary outcome was the immediate post-intervention OSCE total score, analyzed as the group mean at the immediate post-intervention assessment.

Administration

The OSCE was administered at two predefined post-allocation time points: immediately after completion of the allocated educational condition and at the 2–4-week reassessment using the same standardized 14-item OSCE framework.

A baseline OSCE was not performed. Accordingly, the prespecified primary outcome represented an immediate post-intervention between-group comparison rather than change from baseline.

The same simulation environment, procedural task, equipment, candidate instructions, checklist, and scoring framework were used for both randomized groups and at both post-intervention assessments.

Assessor Blinding and Scoring

OSCE performance was evaluated by two trained faculty assessors blinded to randomized group allocation. The assessors used the same 14-item checklist

and item-specific scoring framework and were not involved in delivery of the allocated educational conditions. Before formal assessment, they reviewed the checklist, expected procedural sequence, observable performance behaviors, and application of the 0–2 scoring criteria.

Inter-rater reliability for OSCE scoring was **ICC = 0.89**. Because the original detailed ICC output was unavailable during manuscript revision, the ICC model, agreement definition, confidence interval, and single- versus average-measure specification could not be verified and were not retrospectively reconstructed.

Relationship to the Simulated Vasovagal Reaction Assessment

The 14-item LP OSCE and simulated vasovagal reaction management checklist were separate assessment instruments with distinct scores. The OSCE provided the prespecified primary procedural-performance outcome, whereas the vasovagal checklist provided a separate secondary outcome.

OSCE Item 12 assessed a general response to patient discomfort or a procedural concern and did not assess the complete vasovagal management sequence. The separate vasovagal assessment evaluated recognition and initial management of the predefined simulated vasovagal event using a 7-item checklist with a total score ranging from 0 to 14. Its score did not contribute to the 0–28 OSCE total, and the two outcomes were analyzed separately.

Procedural Performance at the 2–4-Week Reassessment

The same standardized 14-item OSCE framework was administered at the 2–4-week reassessment. A between-group difference in OSCE procedural performance was observed at reassessment; however, the group × time interaction was not statistically significant ($\beta = 0.38$ points; 95% CI, −1.67 to 2.44; $p = 0.714$). The reassessment therefore represents procedural performance at 2–4 weeks rather than evidence of differential skill retention.

Because the same OSCE framework was used at the immediate and 2–4-week assessments, testing or recall effects associated with repeated exposure to the assessment framework cannot be excluded.

Interpretation

The total OSCE score represented directly observed performance on the LP procedural behaviors specified in the 14-item checklist within a standardized simulation environment.

The OSCE did not assess patient-based LP performance. Accordingly, higher OSCE scores should not be interpreted as evidence of independent clinical

competence, transfer to patient-based LP performance, or improved patient safety.

The ultrasound components of the OSCE assessed preprocedural ultrasound localization within the simulated task and did not evaluate real-time ultrasound visualization or guidance of needle advancement.

Instrument Development and Pilot Testing

The OSCE framework underwent expert content review and pilot testing before participant enrollment as part of the study instrument-development process. Expert review addressed item relevance, clarity, suitability for clinical-year medical students, alignment with the intended procedural-learning objectives, and application of the scoring framework.

Pilot testing evaluated feasibility, administration, scenario flow, equipment requirements, and application of the scoring criteria. Clarifications identified during this process were incorporated before participant enrollment.

Complete quantitative content-validity estimates were unavailable in the retained study records. Unsupported values were therefore not retrospectively reconstructed or estimated.

Reporting Note

The item-specific behavioral anchors presented in this supplementary table provide operational descriptions of the original 14-item, 0–2 scoring framework and are included to improve transparency regarding application of the scoring criteria. They do not alter the original number of items, item weighting, total score range, outcome definition, assessment time points, or participant-level scores used in the statistical analyses.

Abbreviations: ICC, intraclass correlation coefficient; LP, lumbar puncture; OSCE, Objective Structured Clinical Examination.

Supplementary Table S5. Standardized Simulated Vasovagal Reaction Management Checklist and Scenario

Purpose

The simulated vasovagal reaction assessment evaluated participants' recognition and initial management of a standardized intraprocedural event during simulated lumbar puncture (LP). The assessment focused on recognition of clinical deterioration, interruption of procedural activity, immediate patient-safety assessment, appropriate positioning, assistance-seeking and escalation,

communication and reassurance, initial supportive management, and reassessment.

The assessment evaluated responses to one predefined simulated vasovagal reaction. It was not designed to assess comprehensive management of LP-related complications or to imply that vasovagal reactions represent the most frequent, most severe, or highest-priority complication associated with LP.

Standardized Clinical Scenario

The vasovagal reaction was incorporated into a standardized simulated LP encounter involving a clinically stable adult patient undergoing diagnostic evaluation for suspected meningitis. Before the candidate entered the station, the simulated patient was considered to have undergone appropriate clinical assessment, with no identified contraindication to LP, and the procedure had been determined appropriate to proceed.

The simulated patient was initially conscious, responsive, and clinically stable. At a predefined stage during the procedure, the patient stated:

"I suddenly feel light-headed and a little nauseated."

The clinical condition then progressed according to the standardized scenario sequence, with the following findings:

- pallor and diaphoresis;
- persistent light-headedness and nausea;
- bradycardia, with a heart rate of approximately 48 beats/min;
- hypotension, with a blood pressure of approximately 85/50 mmHg; and
- continued responsiveness unless further deterioration occurred according to the predefined scenario sequence.

Facilitators provided clinical information according to the predefined progression of the scenario but did not identify the diagnosis or direct participants toward a particular management action. Participants were expected to recognize the change in clinical condition, interrupt procedural activity when appropriate, assess the patient, position the patient safely, seek assistance, communicate with and reassure the patient, initiate appropriate supportive management, and reassess the patient's response.

Standardized Simulated Vasovagal Reaction Management Checklist

Item	Assessed behavior	0 points	1 point	2 points
1	Recognition of clinical deterioration	Does not recognize the clinical deterioration or substantially misinterprets the findings.	Recognizes that deterioration is occurring, but recognition is delayed, incomplete, or requires prompting.	Promptly recognizes the clinical deterioration and identifies the findings as consistent with a vasovagal reaction.
2	Interruption of procedural activity	Continues procedural or needle-related activity despite the patient's deterioration.	Pauses or stops the procedure only after delay or prompting.	Promptly pauses or stops procedural activity and maintains procedural safety.
3	Immediate patient-safety assessment	Does not assess the patient's immediate clinical condition.	Performs an incomplete assessment or requires prompting.	Promptly assesses the patient's immediate condition, including consciousness and relevant airway, breathing, circulation, and vital-sign parameters.
4	Appropriate patient positioning	Leaves the patient in an inappropriate or potentially unsafe position.	Repositions the patient incompletely, after delay, or with prompting.	Promptly places the patient in an appropriate safe position according to the simulated clinical condition.
5	Assistance-seeking and escalation	Does not seek assistance despite clinical deterioration.	Seeks assistance only after delay or prompting, or communicates the situation incompletely.	Promptly seeks appropriate assistance and clearly communicates the patient's clinical deterioration.
6	Communication and reassurance	Does not communicate appropriately with or reassure the patient.	Provides limited, delayed, or inconsistent communication and reassurance.	Maintains clear communication, explains immediate actions, and provides appropriate reassurance throughout the event.
7	Initial supportive management and reassessment	Does not initiate appropriate supportive management or reassess the patient.	Initiates some appropriate measures, but management or reassessment is incomplete or requires prompting.	Initiates appropriate supportive management, monitors the patient's response, and reassesses clinical stability before considering further procedural activity.

The behavioral anchors operationalize the seven predefined assessment domains using the 0–2 scoring framework. Their presentation in this supplementary table is intended to clarify application of the scoring criteria and does not alter the item structure, total score range, outcome definition, or participant-level scores used in the analyses.

Scoring

Each of the seven checklist items was scored from 0 to 2 points:

- 0 points: the assessed behavior was absent, inappropriate, or unsafe;
- 1 point: the behavior was partially or incompletely performed, delayed, or required prompting or correction; and
- 2 points: the behavior was performed appropriately and independently within the standardized simulated scenario.

All seven items were equally weighted. Item scores were summed to generate a total score ranging from 0 to 14, with higher scores representing better performance on the behaviors assessed during the standardized simulated vasovagal event.

This outcome was analyzed separately as an exploratory secondary outcome.

Administration and Scenario Standardization

The simulated vasovagal reaction management assessment was administered immediately after completion of the allocated educational condition. A baseline assessment of this outcome was not performed.

Both randomized groups were assessed using the same standardized scenario, physiological findings, facilitator cues, checklist, and scoring framework under simulation-center conditions.

The initial symptom cue and subsequent clinical findings were predefined to reduce variation in scenario delivery. Facilitators provided clinical information required for progression of the scenario but did not provide corrective feedback, diagnostic suggestions, or management instructions during the scored assessment. When participants appropriately requested additional clinical information, the corresponding standardized findings were provided.

Because this outcome was assessed only after the educational conditions, it represents an immediate post-intervention between-group comparison rather than change from baseline.

Relationship to the LP Procedural-Performance OSCE

The simulated vasovagal reaction management assessment and the 14-item LP procedural-performance OSCE were separate outcome measures with separate scoring instruments.

The LP OSCE evaluated simulation-based LP procedural performance using 14 items and generated a total score ranging from 0 to 28. The vasovagal reaction checklist specifically evaluated recognition and initial management of the predefined simulated event using seven items and generated a separate total score ranging from 0 to 14.

Although both instruments included patient-safety and communication behaviors, their assessment domains differed. OSCE Item 12 addressed the participant's general response to patient discomfort or a procedural concern, whereas the vasovagal checklist provided a focused assessment of responses to the standardized vasovagal event. Consistent with the OSCE supplementary material, the vasovagal score did not contribute to the 0–28 OSCE total.

The two outcomes were therefore scored and analyzed separately.

Scope and Interpretation

The total checklist score represented performance in recognizing and initially managing one standardized simulated vasovagal reaction within the study environment.

The assessment did not evaluate comprehensive LP complication management. In particular, it did not assess participants' management of other potential LP-related events or complications, such as traumatic puncture, post-dural puncture headache, bleeding, infection, or neurological complications. Patient selection, contraindications, and broader procedural-safety knowledge were addressed elsewhere within the educational program and assessment framework.

The vasovagal assessment was also closely aligned with content included in the UIS-LP program and was administered only after the educational conditions. Higher scores therefore represent performance on the specific standardized simulated task assessed and should not be extrapolated to unfamiliar procedural events, other LP-related complications, or patient-based clinical care.

Accordingly, performance on this assessment should not be interpreted as evidence of independent clinical competence, comprehensive complication-management ability, or improved patient safety.

Rationale for Selection of the Vasovagal Scenario

The vasovagal reaction was selected as a standardized intraprocedural event suitable for incorporation into the simulation-based educational program. The scenario permitted assessment of observable responses to evolving clinical deterioration, including recognition of the event, interruption of procedural activity, immediate patient-safety assessment, positioning, communication and reassurance, assistance-seeking, supportive management, and reassessment.

Its inclusion extended the simulated encounter beyond technical execution of LP by incorporating recognition and initial management of an unexpected intraprocedural event.

Importantly, selection of the vasovagal scenario was not based on an assumption that it represents the most frequent, most severe, or highest-priority complication of LP. The scenario was not intended to replace education concerning appropriate patient selection, contraindications, or other procedure-related complications. Evaluation of responses to a broader range of procedural events would require additional scenarios and was outside the scope of the present trial.

Instrument Development, Content Review, and Pilot Testing

The simulated vasovagal reaction management checklist underwent expert content review and pilot testing before participant enrollment as part of the study instrument-development process. Expert review addressed item relevance, clarity, suitability for clinical-year medical students, alignment with the intended educational domains, scenario flow, and application of the scoring framework.

Pilot testing evaluated clarity, feasibility, administration, scenario progression, facilitator cues, equipment requirements, and application of the scoring criteria. Clarifications identified during this process were incorporated before participant enrollment.

Complete quantitative content-validity estimates and a separate reliability estimate for the 7-item checklist were unavailable in the retained study records. Unsupported estimates were therefore not retrospectively reconstructed or calculated.

Reporting Note

The item-specific behavioral anchors presented in Supplementary Table S5 operationalize the original 7-item, 0–2 scoring framework and do not alter the number of items, item weighting, total score range, outcome definition, or participant-level scores used in the analyses.

Because the assessment was administered only post-intervention, was closely aligned with UIS-LP content, and evaluated one predefined simulated event, its findings represent performance on the specified assessment rather than generalized complication-management competence or transfer to clinical practice.

Assessment Timing and Sequence

Assessment Schedule

Study assessments were conducted at three predefined time points: baseline, immediately after completion of the allocated educational condition, and at the 2–4-week reassessment. At corresponding time points, both randomized groups were evaluated using the same applicable instruments and scoring procedures under standardized study conditions.

Baseline Assessment

Before randomization, participants completed the 9-item Procedural Knowledge Assessment (0–9) and 10-item Self-Confidence Questionnaire (10–50). Demographic and educational characteristics, including previous LP exposure, were also recorded.

LP procedural performance was not assessed by OSCE at baseline. Ultrasound sonoanatomy identification and simulated vasovagal reaction management were also not assessed at baseline. Consequently, baseline comparability on the primary procedural-performance outcome could not be directly evaluated, and the primary outcome was analyzed using the immediate post-intervention OSCE score rather than change from baseline.

Immediate Post-Intervention Assessment

Immediately after completion of the allocated educational condition, participants completed the following assessments:

1. **LP Procedural-Performance OSCE:** 14-item checklist; score range, 0–28.
2. **Procedural Knowledge Assessment:** 9-item assessment; score range, 0–9.
3. **Ultrasound Sonoanatomy Identification Assessment:** 10-item image-based assessment; score range, 0–10.
4. **Self-Confidence Questionnaire:** 10-item self-reported measure; score range, 10–50.
5. **Simulated Vasovagal Reaction Management Assessment:** 7-item checklist; score range, 0–14.

The applicable instruments and scoring procedures were identical for both randomized groups. Cognitive and self-reported measures were administered separately from simulation-based performance assessments.

The LP procedural-performance OSCE and simulated vasovagal reaction management assessment were distinct outcomes with separate scoring instruments. The vasovagal score did not contribute to the 0–28 OSCE total.

The participant-level sequence of the immediate assessments was not retained; therefore, a fixed assessment order is not reported.

2–4-Week Reassessment

LP procedural performance was reassessed within the prespecified 2–4-week window using the same 14-item OSCE and 0–28 scoring framework. The procedural task, simulation environment, equipment, candidate instructions, checklist, and scoring procedures were standardized across the immediate and reassessment time points.

Procedural knowledge, ultrasound sonoanatomy identification, self-confidence, and simulated vasovagal reaction management were not reassessed at 2–4 weeks.

Because the same OSCE framework was used at both post-intervention assessments, testing or recall effects cannot be excluded. The delayed assessment is therefore reported as **LP procedural performance at the 2–4-week reassessment** rather than evidence of differential skill retention.

Follow-Up Interval

All 78 randomized participants completed the OSCE reassessment within the prespecified 2–4-week window. Exact participant-level reassessment intervals were not retained; therefore, the number of days between assessments, its distribution by randomized group, and potential effects of variation in reassessment timing could not be evaluated. No mean or median interval is reported because these values could not be verified from the retained study records.

Summary of Assessment Timing

Assessment	Baseline	Immediate Post-Intervention	2–4-Week Reassessment
Procedural knowledge (0–9)	Yes	Yes	No
Self-confidence (10–50)	Yes	Yes	No

Assessment	Baseline	Immediate Post-Intervention	2–4-Week Reassessment
Ultrasound sonoanatomy identification (0–10)	No	Yes	No
LP procedural-performance OSCE (0–28)	No	Yes	Yes
Simulated vasovagal reaction management (0–14)	No	Yes	No

Interpretation of Assessment Timing

The assessment schedule is important when interpreting the study outcomes. Because LP procedural performance was not assessed at baseline, baseline comparability on the primary OSCE outcome could not be directly established. Baseline procedural knowledge was a cognitive measure and should not be considered a surrogate for directly observed procedural performance. The prespecified primary analysis therefore compared immediate post-intervention OSCE scores between groups; adjustment for baseline procedural knowledge was reported separately as a post hoc sensitivity analysis.

Ultrasound sonoanatomy identification and simulated vasovagal reaction management were assessed only post-intervention. These outcomes therefore represent immediate between-group comparisons rather than change from baseline and should be interpreted as performance on the specified assessment tasks.

At the 2–4-week reassessment, a between-group difference in OSCE procedural performance was observed, whereas the group × time interaction was not statistically significant. The reassessment therefore documents procedural performance at 2–4 weeks but does not establish a different pattern of change between groups or differential skill retention. Repeated use of the same OSCE framework may also have introduced testing or recall effects.

Content Validity, Pilot Testing, and Inter-Rater Reliability

Instrument Development and Content Review

The study assessment framework comprised the procedural knowledge assessment, self-confidence questionnaire, ultrasound sonoanatomy identification assessment, LP procedural-performance OSCE, and simulated vasovagal reaction management checklist. The instruments were developed or

adapted to align with the predefined learning objectives and assessment domains.

Before participant enrollment, the instruments underwent expert content review by individuals with relevant experience in anesthesiology, medical education, simulation-based education, procedural-skills training, and ultrasound-assisted procedures. Review addressed item relevance, clarity, suitability for clinical-year medical students, and alignment with the intended assessment domains. For performance-based measures, reviewers also considered suitability of the assessed behaviors for standardized simulation-based evaluation.

Content-Validity Evidence

Where quantitative validation records were available, content validity was evaluated using the Content Validity Index (CVI). Item relevance was rated on a 4-point scale (1 = not relevant, 2 = somewhat relevant, 3 = quite relevant, and 4 = highly relevant). The item-level Content Validity Index (I-CVI) represented the proportion of experts assigning a relevance rating of 3 or 4, and the scale-level Content Validity Index using the average method (S-CVI/Ave) represented the mean I-CVI across items.

For the 10-item self-confidence questionnaire, the retained records documented an **S-CVI/Ave of 0.96** and internal consistency of **Cronbach's α = 0.81**.

Complete item-level I-CVI records and instrument-specific quantitative validity estimates were unavailable for all other assessment instruments. Unsupported values were therefore not retrospectively reconstructed or estimated.

Instrument Finalization and Pilot Testing

Comments arising from expert review were considered before instrument finalization, with wording and instructions clarified where necessary. Final item structures, scoring ranges, and administration procedures were established before participant enrollment.

Before formal study implementation, the assessment instruments and study procedures were pilot tested with eight clinical-year medical students who were not included in the final study sample. Pilot testing evaluated clarity, feasibility, administration time, scenario flow, equipment requirements, and application of the scoring framework. Minor clarifications to wording, instructions, and assessment procedures were incorporated before participant enrollment and formal data collection.

The item-specific behavioral anchors presented in Supplementary Tables S4 and S5 provide operational descriptions of the existing 0–2 scoring frameworks and

do not alter the number of items, score ranges, item weighting, outcome definitions, or participant-level scores used in the analyses.

OSCE Assessor Procedures and Calibration

LP procedural performance was evaluated by two faculty assessors blinded to randomized group allocation. Both assessors used the same 14-item OSCE checklist and scoring framework and were not involved in recruitment, randomization, or delivery of the allocated educational conditions.

Before formal outcome assessment, the assessors reviewed the checklist, expected procedural sequence, observable performance behaviors, and application of the 0–2 scoring criteria.

Inter-Rater Reliability

Inter-rater reliability for OSCE procedural-performance scoring was **ICC = 0.89**.

The retained analytical dataset contained one participant-level OSCE score at each assessment time point rather than separate assessor-level scores. Because the original detailed ICC output was unavailable during manuscript revision, the ICC model, agreement definition, confidence interval, and single- versus average-measure specification could not be verified and were not retrospectively reconstructed.

The available records also did not permit verification of how the two assessor ratings were combined to generate the retained participant-level score. Accordingly, the final score is not retrospectively characterized as an average, consensus score, or another specific combination method, and no participant-level scores were recalculated or modified during manuscript revision.

Interpretation of the Measurement Evidence

The available content-validity evidence supports the relevance of the assessment content to the educational domains evaluated in this study but should not be interpreted as evidence of comprehensive construct or predictive validity, independent clinical competence, or transfer of simulation-based performance to patient care.

The reported ICC = 0.89 relates specifically to inter-rater reliability of OSCE procedural-performance scoring within the standardized simulation environment. The unavailable ICC specifications limit more detailed characterization of the reliability analysis.

Educational Harms and Unintended Events

Monitoring and Safety Procedures

Educational and simulation-related unintended events were monitored during supervised study activities and assessments. Monitoring included physical discomfort, distress or anxiety requiring interruption, equipment-related incidents, withdrawal or discontinuation related to study activities, medical evaluation, or modification of planned study procedures.

Participants could pause or discontinue an educational or assessment activity if they experienced discomfort or wished to stop. Faculty members and simulation-center personnel were present during supervised activities to address immediate concerns.

All needle-insertion practice was performed on the designated LP simulator. No study-related invasive procedure was performed on participants or patients. The standardized vasovagal reaction was a scripted simulated event using predefined clinical cues and simulated physiological findings; no physiological deterioration was induced.

Potential Unintended Educational Effects

Potential unintended effects included discomfort or anxiety associated with observed procedural assessment and frustration during simulated procedural practice. Participants were instructed not to share study-specific educational materials or assessment content across randomized groups before completion of study assessments. Cross-group contamination was not formally assessed, and additional LP- or ultrasound-related educational exposure outside the allocated conditions was not systematically recorded.

Differences in educational exposure between UIS-LP and SDL were features of the study design rather than unintended events. UIS-LP included instructor-led teaching, supervised ultrasound localization, hands-on simulation, repeated practice, feedback, and debriefing, whereas SDL did not include equivalent instructor contact or hands-on procedural exposure.

Observed Harms and Unintended Events

No educational or simulation-related unintended events meeting the predefined monitoring criteria were documented during the intervention or assessment sessions. No event requiring medical evaluation, participant withdrawal, discontinuation of training or assessment, or modification of planned study procedures was recorded, and no equipment-related incident resulting in participant harm was documented.

All 78 randomized participants completed the immediate assessments and 2–4-week OSCE reassessment, with no loss to follow-up attributable to a documented educational or simulation-related unintended event.

A dedicated instrument or formal surveillance system was not used to quantify transient anxiety, psychological distress, discomfort, or other minor subjective experiences. The absence of documented events therefore should not be interpreted as evidence that no participant experienced such transient effects.

Interpretation

These safety observations apply only to the educational and simulation-based activities evaluated in this trial and do not provide evidence regarding the safety of patient-based LP or ultrasound-assisted LP in clinical practice.

The study was not designed or powered to identify uncommon educational or psychological adverse effects, and minor subjective effects were not systematically measured using dedicated instruments. Their frequency or severity therefore cannot be determined from this study.

Reporting Statement

No educational or simulation-related unintended events meeting the predefined monitoring criteria were documented during the study. No event requiring medical evaluation, participant withdrawal, discontinuation of training or assessment, or modification of planned study procedures was recorded.
